# Defective targeting of PNPLA1 to lipid droplets causes ichthyosis in ABHD5-syndromic epidermal differentiation disorder

**DOI:** 10.1016/j.jlr.2025.100875

**Published:** 2025-08-14

**Authors:** Margarita Schratter, David Holubek, Lukas Koeffler, Thomas Züllig, Thomas O. Eichmann, Heimo Wolinski, Monika Oberer, Achim Lass, Franz P.W. Radner

**Affiliations:** 1 Institute of Molecular Biosciences, University of Graz, Graz, Austria; 2 Core Facility Mass Spectrometry, ZMF, Medical University of Graz, Graz, Austria; 3 BioTechMed-Graz, Graz, Austria; 4 Field of Excellence BioHealth, University of Graz, Graz, Austria

**Keywords:** skin, sphingolipids, ceramides, acylceramides, triacylglycerol, disease mechanisms, enzymology/enzyme mechanisms, neutral lipid storage disease, fluorescence microscopy

## Abstract

ABHD5-syndromic epidermal differentiation disorder (ABHD5-sEDD; also known as Chanarin-Dorfman syndrome) is a rare autosomal recessive disorder caused by mutations in the α/β-hydrolase domain-containing 5 (*ABHD5*) gene, leading to systemic accumulation of neutral lipids and ichthyosis due to impaired activation of patatin-like phospholipase domain-containing (PNPLAs) proteins. While ABHD5 is a well-known co-activator of adipose triglyceride lipase (ATGL, also referred to as PNPLA2), its role in epidermal lipid metabolism is incompletely understood. Here, we identify ABHD5 as a key regulator of PNPLA1, an enzyme essential for ω-*O*-acylceramide (acylCer) synthesis and skin barrier formation. We analyzed seven disease-associated *ABHD5* missense mutations and found that they disrupt PNPLA1 localization and function by distinct mechanisms: (i) mutations affecting the PNPLA1 binding region of ABHD5 impair PNPLA1 recruitment to intracellular lipid droplets (LDs), thus reducing acylCer synthesis; (ii) mutations in potential perilipin-binding domains of ABHD5 prevent ABHD5 association with LDs, thereby disrupting PNPLA1-LD localization. Despite these defects, restoring co-localization of ABHD5 mutants with PNPLA1 in proteoliposomes rescued full PNPLA1 enzyme activity, indicating that spatial proximity rather than direct protein binding is sufficient to facilitate acylCer formation. In summary, our findings establish a co-localization-driven model of PNPLA1 regulation, in which ABHD5 ensures proper PNPLA1 targeting to LDs and simultaneously enables its enzymatic activation. This model suggests that pharmacological strategies aimed at restoring PNPLA1 localization to LDs may represent a potential therapeutic approach for ichthyosis in ABHD5-sEDD. By elucidating the molecular mechanisms underlying disease pathogenesis, our study provides important new insights into epidermal lipid metabolism and therapeutic targeting.

ABHD5-syndromic epidermal differentiation disorder (ABHD5-sEDD; also known as Chanarin-Dorfman syndrome) is a rare and severe autosomal recessive disorder that belongs to the group of Neutral Lipid Storage Diseases (1, 2, 3). It arises from mutations in the *ABHD5* gene, which encodes the α/β-hydrolase domain-containing 5 protein (also known as comparative gene identification-58, CGI-58) (4). Human ABHD5 is a 349-amino acid protein (Fig. 1A), localizing to lipid droplets (LDs). It plays a crucial role in lipolysis by activating adipose triglyceride lipase (ATGL) – also known as patatin-like phospholipase domain-containing 2 (PNPLA2) – the key enzyme responsible for initiating the breakdown of intracellular triacylglycerol (TAG) stores (5). In the absence of functional ABHD5, TAGs accumulate to pathophysiological levels across virtually every cell type, leading to a wide range of clinical symptoms, including hepatomegaly, myopathy, developmental delays, and neurological impairments, such as sensorineural hearing loss or mental retardation (1, 2, 3, 6).

**Fig. 1:**
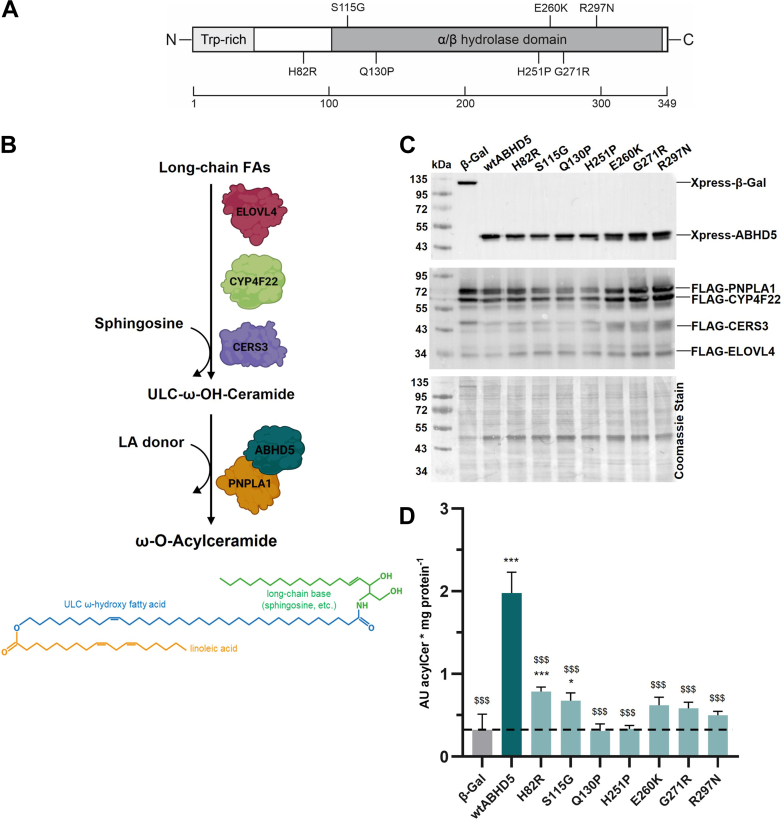
Disease-associated ABHD5 mutants are defective in stimulating PNPLA1-mediated acylCer synthesis. A: Schematic representation of the human ABHD5 protein structure, highlighting ABHD5-sEDD-associated point mutations. B: Proposed biosynthesis pathway and molecular structure of acylCer. Schematic representation was created using BioRender.com. The synthesis pathway starts with long-chain fatty acids, which are elongated by the fatty acid elongase ELOVL4 to generate ultra-long chain (ULC) fatty acids that are subsequently ω-hydroxylated by the cytochrome P450 monooxygenase CYP4F22. The resulting ULC-ω-OH fatty acids are then N-acylated to sphingoid bases by ceramide synthase 3 (CERS3), producing ULC-ω-OH-ceramides. In the final step, PNPLA1 catalyzes the transacylation of linoleic acid (LA) from a donor molecule onto these ceramides, forming acylCers. This reaction is stimulated by the co-activator ABHD5. C: Immunoblot analysis of co-expression of tagged human fusion proteins CERS3, CYP4F22, ELOVL4, PNPLA1, and different ABHD5 mutants or β-galactosidase (β-Gal) in HEK293T cells using respective antibodies. D: Analysis of acylCer biosynthesis in HEK293T cells co-expressing human CERS3, CYP4F22, ELOVL4, PNPLA1, and different ABHD5 variants or β-Gal using targeted UHPLC-MS/QQQ. Dashed line indicates basal PNPLA1-mediated acylCer formation. Data are presented as means of triplicates + SD and are representative of three independent experiments. Statistically significant differences were determined using one-way ANOVA (∗, *P* < 0.05; ∗∗∗, *P* < 0.001 compared to the negative control β-Gal and $, *P* < 0.05; $$, *P* < 0.01; $$$, *P* < 0.001 compared to wtABHD5). Abbreviations: ABHD5, α/β-hydrolase domain-containing 5; acylCer, ω-*O*-acylceramide; AU, arbitrary units; PNPLA1, patatin-like phospholipase domain-containing 1; wt, wild-type; ω-OH, ω-hydroxy.

The most defining clinical feature of ABHD5 deficiency, however, is the invariable onset of congenital ichthyosis, a severe skin disorder marked by dry, thickened skin with abnormal scaling, often involving the entire body surface (7). This condition is closely associated with impaired sphingolipid metabolism in the epidermis, resulting in compromised skin integrity and barrier function (4, 8). Lipidomic analyses have revealed that individuals with *ABHD5* mutations are severely deficient in ω-*O*-acylceramides (acylCers), a crucial lipid species for maintaining the epidermal permeability barrier (Fig. 1B) (8, 9, 10, 11). Instead, ω-OH-ceramides, which are precursors of acylCers lacking esterified linoleic acid at the ω-OH position of the ultra-long chain fatty acid, accumulate massively. This defect in acylCer formation leads to a compromised skin barrier, which is associated with an increased transepidermal water loss and rapid dehydration. Interestingly, a strikingly similar skin phenotype is observed in both patients and mouse models with mutations in the *PNPLA1* gene (12, 13, 14, 15). This significant observation has led to the conclusion that ABHD5 and PNPLA1 are functionally linked, operating within the same epidermal lipid biosynthetic pathway (13, 14, 16).

At the core of this functional interaction, PNPLA1 catalyzes the transacylation of ω-OH-ceramides with LA to generate acylCers (Fig. 1B), a process essential for an intact skin barrier (17, 18, 19). However, the ability of PNPLA1 to perform this reaction largely depends on the presence of ABHD5 as a co-activator, making this functional interaction central to lipid metabolism in the skin. These two proteins physically interact and co-localize on the surface of LDs, where stored neutral lipids, like linoleic acid-containing TAGs, serve as acyl-donor substrates for PNPLA1 transacylase activity (11).

Crucially, perilipin (PLIN) proteins, which coat LDs, regulate the accessibility of enzymes such as ATGL (PNPLA2), PNPLA1, or PNPLA3 to these lipid depots. In this process, ABHD5 also interacts with PLINs, controlling the proper localization of ABHD5 to the LD surface, and thereby regulating intracellular TAG mobilization (20, 21). In ABHD5-sEDD patients, however, mutations in *ABHD5* have been shown to impair the ability of ABHD5 to localize correctly to LDs by disrupting the crucial protein interactions with PLINs (22).

Despite the known interactions between ABHD5, PLINs, and PNPLA proteins, the exact molecular mechanism by which ABHD5 stimulates PNPLA1 or the enzymatic activities of other PNPLA proteins remains poorly understood. Furthermore, while several disease-associated *ABHD5* mutations have been characterized, their impact on the co-localization of ABHD5 with PNPLA1 or PLINs at the surface of LDs has not been systematically investigated (22). Here, we explore this critical gap in understanding epidermal lipid metabolism by examining frequent ABHD5 point mutants (Fig. 1A) to decipher the molecular mechanism underlying ichthyosis pathogenesis in ABHD5-sEDD (4, 22, 23, 24, 25, 26, 27, 28, 29, 30). Our findings not only provide new insights into the lipid metabolic dysfunctions in the skin but also enhance our knowledge of the pathophysiological processes contributing to other systemic manifestations observed in this disorder.

## Materials and methods

### Cell culture and transfection

HEK293T (ATCC CRL-3216) or COS-7 (ATCC CRL-1651) cells were maintained in DMEM (Thermo Fisher Scientific, Waltham, MA) supplemented with 10% FBS (Sigma-Aldrich, St. Louis, MO), 100 IU/ml penicillin (Thermo Fisher Scientific), and 100 μg/ml streptomycin (Thermo Fisher Scientific) at 37°C in a humidified 7% CO_2_ atmosphere. Cells were transfected with plasmid DNA using either Metafectene (Biontex GmbH, Munich, Germany) or Lipofectamine 3000 Reagent (Thermo Fisher Scientific) according to the manufacturer’s guidelines. Expi293F cells (Thermo Fisher Scientific) were cultured in Expi293 Expression Medium (Thermo Fisher Scientific) in 125 ml Erlenmeyer flasks at 125 rpm and 37°C in a humidified 7% CO_2_ atmosphere, and passaged every 3–4 days upon reaching 3-5 × 10^6^ viable cells/ml. Transfection was performed using the ExpiFectamin 293 Transfection Kit (Thermo Fisher Scientific) according to the manufacturer’s protocol. Primary human keratinocytes from skin biopsies were prepared, cultured, and terminally differentiated *in vitro* as described previously (31).

### RNA isolation and reverse transcription PCR

Total RNA was isolated from HEK293T cells or primary human keratinocytes at day 14 of differentiation using TRIzol Reagent (Thermo Fisher Scientific) according to the manufacturer’s instructions. One microgram of RNA was reverse-transcribed into cDNA using the PrimeScript™ High Fidelity RT-PCR Kit (Takara Bio USA, San Jose, CA). Gene-specific primer pairs (listed in Supplemental Table S1) were used for PCR amplification, and the resulting cDNA products were used for subsequent molecular cloning.

### Molecular cloning of recombinant proteins

Full-length open reading frames of *ABHD5* (NM_016006.6), *PNPLA1* (NM_001145717.1), *PLIN2* (NM_001122.4), or *PLIN3* (NM_005817.5) were amplified by PCR from human cDNA using Q5 DNA Polymerase (New England Biolabs, NEB, Ipswich, MA, USA) and gene-specific primers containing endonuclease cleavage sites (Supplemental Table S1). PCR products were cloned into expression vectors pcDNA4/HisMaxC (Thermo Fisher Scientific), pcDNA4/HisMaxC-mut (see below), pEYFP-C1, or pECFP-C1 (both from Takara Bio USA) via compatible restriction sites. A control pcDNA4/HisMaxC vector encoding β-galactosidase (β-Gal) was provided by the manufacturer (Thermo Fisher Scientific). The generation of pFLAG-CMV-hsPNPLA1 and the multicistronic lentiviral plasmid pLVX-ultra-IRES-Puro, encoding ceramide synthase 3 (*CERS3,* NM_178842.4), cytochrome P450 family 4 subfamily F member 22 (*CYP4F22,* NM_173483.4), and FA elongase-4 (*ELOVL4,* NM_022726.4), has been described previously (18). To obtain a bicistronic *CERS3*-*ELOVL4* construct, *CYP4F22* was removed from the multiple cloning site (MCS) II of pLVX-ultra-CERS3-CYP4F22-ELOVL4-IRES-Puro by AgeI (NEB) restriction digestion, and the remaining fragment was re-ligated. For a bicistronic *PNPLA1*-*CYP4F22* construct, the *PNPLA1* coding sequence was amplified from human cDNA using gene-specific primers introducing SpeI (NEB) cleavage sites, and inserted into MCSI of pLVX-ultra-IRES-Puro (18). *CYP4F22* coding sequence was excised from pLVX-ultra-IRES-Puro-CERS3-CYP4F22-ELOVL4 using NotI-HF (NEB) and XhoI (NEB) and cloned into MCSII of the PNPLA1-containing vector via compatible restriction sites. For expression of 3x-FLAG-tagged PNPLA1, ABHD5, or T1R1, respective coding sequences were PCR-amplified using Q5 DNA Polymerase (NEB) and gene-specific primer pairs (Supplemental Table S1), either from human cDNA or the provided control plasmid pEU-E01-T1R1 (CellFree Sciences, Matsuyama, Japan), and initially inserted into MCSI or MCSIII of the pLVX-ultra-IRES-Puro vector. 3x-FLAG-tagged sequences were then re-amplified using corresponding primer pairs (Supplemental Table S1) and subcloned into the MCS of the pEU-E01 vector (CellFree Sciences) or the pcDNA4/HisMaxC-mut (see below) for use in cell-free protein synthesis or protein purification.

### Mutagenesis and sequence analysis

Point-mutations in *ABHD5* (p.H82R, p.S115G, p.Q130P, p.H251P, p.E260K, p.G271R, and p.R297N) and *PNPLA1* (p.S53L) were generated using the Q5 Site-Directed Mutagenesis Kit (NEB) with primer pairs listed in Supplemental Table S2. For recombinant protein purification of PNPLA1, PLIN2, and PLIN3, the pcDNA4/HisMaxC expression vector (Thermo Fisher Scientific) was modified using respective primers (Supplemental Table S2) and the same kit to introduce a nucleotide substitution (CTG > TAC) at vector position 1152–1154. This mutation replaces leucine with tyrosine within the Xpress tag, resulting in an altered epitope (Asp-Tyr-Tyr) that is not recognized by the anti-Xpress antibody. This modification was essential to prevent interference with immunodetection during subsequent solid-phase interaction assays. Sequence analyses of generated constructs were performed at Microsynth AG (Balgach, Switzerland) using respective primers.

### Cell-based acylCer biosynthesis assay

To assess acylCer formation *in vivo* in the presence of various ABHD5 variants, 1 × 10^6^ HEK293T cells were seeded in 6-well cell culture dishes pre-coated with 0.1% gelatin (Sigma-Aldrich) and co-transfected with 0.75 μg each of the bicistronic plasmids pLVX-ultra-CYP4F22-PNPLA1-IRES-Puro and pLVX-ultra-CERS3-ELOVL4-IRES-Puro, together with 1 μg of pcDNA4/HisMaxC encoding either wild-type or mutant ABHD5, or β-Gal as a control. Transfection was performed using Metafectene reagent (Biontex) according to the manufacturer’s protocol. After 24 h, the medium was replaced with DMEM supplemented with 10% FBS (Sigma-Aldrich), 100 IU/ml penicillin (Thermo Fisher Scientific), 100 μg/ml streptomycin (Thermo Fisher Scientific), 2 μM sphingosine (Abcam, Cambridge, United Kingdom; dissolved in DMSO), 100 μM nervonic acid (Larodan AB, Solna, Sweden; dissolved in ethanol), and 100 μM linoleic acid (Sigma-Aldrich; dissolved in ethanol). Cells were incubated for 6 h at 37°C, then harvested and washed three times with ice-cold PBS (10 mM Na_2_HPO_4,_ 1.8 mM KH_2_PO_4_, pH 7.4; 140 mM NaCl; 2.7 mM KCl). An aliquot of each cell suspension was used for immunoblotting to confirm protein expression.

For total lipid extraction, cells were incubated with 1 ml methyl-tert-butyl ether (MTBE)/methanol (3/1, v/v) containing 500 nmol butylated hydroxytoluene (BHT), 0.1% acetic acid, and internal lipid standards, under constant shaking for 30 min at room temperature. After addition of 200 μl ddH_2_O and vortexing for 10 s, phase separation was achieved by centrifugation at 10,000 × g for 10 min. The upper organic phase (700 μl) was collected and dried under a stream of nitrogen. Lipids were reconstituted in 150 μl 2-propanol/methanol/ddH_2_O (70/25/10, v/v/v), and 5 μl were subjected to targeted ultra-high performance liquid chromatography-triple quadrupole mass spectrometry (UHPLC-MS/QQQ) for quantification of cellular acylCer levels. Remaining cell pellets were dried and solubilized in 1.2 ml 0.3 M NaOH containing 0.1% SDS overnight at 25°C with constant agitation. Protein concentrations were determined using the Pierce BCA Protein Assay Kit (Thermo Fisher Scientific) and BSA as standard according to the manufacturer’s instructions.

### Lipid analysis by UHPLC-MS/QQQ

Chromatographic separation with a UHPLC (Agilent Infinity II, Santa Clara, CA, USA) was performed on a reverse-phase column (ACQUITY UPLC BEH C18 column; particle size 1.7 μm; inner diameter 2.1 mm; length 150 mm; Waters, Milford, MA, USA) using a 30 min linear gradient from 50% solvent A (ddH_2_O with 10 mM ammonium acetate, 0.1% formic acid, and 8 μM phosphoric acid) to 100% solvent B (2-propanol containing the same additives). The column temperature was maintained at 50°C. Lipids were detected using a triple quadrupole mass spectrometer (Agilent 6470, Santa Clara, CA, USA) equipped with an ESI source operated in positive ion mode with multiple reaction monitoring (MRM) of specific precursor-to-product ion transitions (Supplemental Table S3). Data acquisition was performed using MassHunter Data Acquisition Software (v10.0 SR1, Agilent), and quantitative analysis was carried out using MassHunter QQQ quantitative analysis software (v10.1, Agilent). Relative lipid quantification was based on normalization to the internal standard acylCer (d18:1/26:0/18:1; Avanti Polar Lipids, Alabaster, AL) and expressed as arbitrary units (AU) per milligram of protein.

### Co-immunoprecipitation assay

To assess protein-protein interactions between PNPLA1 and ABHD5 variants or β-Gal as a negative control *in vivo*, 6 × 10^6^ HEK293T cells were seeded in 100-mm cell culture dishes pre-coated with 0.1% gelatin (Sigma-Aldrich) and co-transfected with 7.5 μg of pFLAG-CMV-hsPNPLA1 and 7.5 μg of either pcDNA4/HisMaxC-hsABHD5 variant or pcDNA4/HisMaxC-β-Gal using Metafectene (Biontex) according to the manufacturer’s instructions. After 24 h, cells were harvested and washed three times with ice-cold PBS. Pellets were lysed in 500 μl of buffer B (50 mM Tris/HCl, pH 7.4; 150 mM NaCl; 1 mM EDTA; 1% NP-40; 20 μg/ml leupeptine; 2 μg/ml antipain; 1 μg/ml pepstatin) for 1 h at 4°C under constant rotation. Cell lysates were centrifuged at 1,000 × g for 10 min at 4°C, and supernatants were collected. Protein concentrations were determined using the Pierce BCA Protein Assay Kit (Thermo Fisher Scientific). For immunoblot analysis of input fractions, 20 μg of total protein were separated by SDS-PAGE and analyzed by immunoblotting. For co-immunoprecipitation, 1 mg of protein lysate was incubated overnight at 4°C with 40 μl anti-FLAG M2-agarose beads (Sigma-Aldrich) under gentle rotation. Beads were then washed eight times with 1 ml buffer B, and bound proteins were eluted by boiling at 95°C for 10 min in 2 × SDS sample buffer (0.1 M Tris/HCl, pH 7.4; 5% (v/v) β-mercaptoethanol; 4% (w/v) SDS; 20% (v/v) glycerin; bromphenol blue). A 15 μl aliquot of the eluate (output fraction) was analyzed by immunoblotting.

### Protein purification of PNPLA1, PLIN2, and PLIN3

Expi293F cells were cultured to a density of 3 × 10^6^ cells/ml and transfected with 25 μg of either pcDNA4/HisMaxC-mut-3xFLAG-hsPNPLA1, pcDNA4/HisMaxC-mut-hsPLIN2, or pcDNA4/HisMaxC-mut-hsPLIN3 using the ExpiFectamine 293F Transfection Kit (Thermo Fisher Scientific) according to the manufacturer's guidelines. Seventy-two hours post-transfection, cells were harvested and subjected to protein purification by immobilized metal ion affinity chromatography (IMAC) using the ÄKTA™ pure chromatography system (Cytiva, Marlborough, MA).

For this purpose, cell pellets were washed three times with ice-cold PBS and lysed in 40 ml of buffer A1 (100 mM potassium phosphate buffer [PPB], pH 7.5; 100 mM potassium chloride buffer (PCB); 10% glycerol; 0.1% NP-40; 30 mM imidazole; 1 mM TCEP (Tris(2-carboxyethyl)phosphin); 10 mM ATP; 10 mM MgCl_2_; 20 μg/ml leupeptine; 2 μg/ml antipain; 1 μg/ml pepstatin). Mechanically disruption was performed using an ULTRA-TURRAX® homogenizer (IKA-Werke GmbH & Co. KG, Staufen, Germany) on ice for 30 s, followed by sonication using a Sonoplus Ultrasonic Homogenizer HD 3100 (Bandelin electronic GmbH, Berlin, Germany; TT13 titanium plate probe) at 45% amplitude (0.5 s on/off pulses for 3 min). A 100 μl aliquot of the homogenates was stored at −20°C for immunoblotting. Lysates were clarified by centrifugation at 40,000 × g for 30 min at 4°C, and a 100 μl aliquot of the supernatant (= soluble lysate fraction) was collected for immunoblot analysis. The remaining pellet was resuspended in 7 ml of buffer A1 and again sonicated (MS72 probe; 20% amplitude; 10 s on/off pulses for 3 min). A 100 μl aliquot of this insoluble fraction was stored at −20°C for immunoblot analysis. The clarified lysate was filtered through a 0.45 μm single-use filter unit (Lab Logistics Group GmbH, Meckenheim, Germany) and loaded onto a 1 ml HisTrap HP column (Cytiva) connected to the ÄKTA™ pure chromatography system.

The method used for protein purification started with column equilibration using 7 column volumes (1 CV = 1 ml) of buffer A1, followed by sample loading and sequential washes: 5 CV of buffer A1, 7 CV of Ni-wash buffer A3 (100 mM PPB, pH 7.5; 100 mM PCB; 10% glycerol; 0.003% NP-40; 30 mM imidazole; 1 mM TCEP; 10 mM ATP; 10 mM MgCl_2_), and 10 CV of equilibration buffer A2 (100 mM PPB, pH 7.5; 100 mM PCB; 10% glycerol; 1 mM TCEP). Final washing was performed with 5 CV of A2 containing 15% elution buffer B1 (composition as A3 but with 500 mM imidazole). Target proteins were eluted using 100% elution buffer B1 in 1 ml fractions, and 100 μl aliquots were stored at −80°C. Protein presence and purity in all collected fractions (homogenate, lysates, insoluble, and eluates) were verified by immunoblot analysis.

### Solid-phase interaction assay

To assess and quantify protein-protein interactions between PNPLA1, PLIN2, or PLIN3 and the respective ABHD5 variants or β-Gal as a negative control, HEK293T cells (6 × 10^6^ per 100-mm dish; pre-coated with 0.1% gelatin; Sigma-Aldrich) were transfected with 15 μg of pcDNA4/HisMaxC constructs encoding ABHD5 variants or β-Gal using Lipofectamine 3000 Reagent (Thermo Fisher Scientific) according to the manufacturer’s protocol. Eight hours post-transfection, the proteasome inhibitor MG-132 (Sigma-Aldrich) was added at a final concentration of 0.7 μM to prevent degradation of overexpressed proteins. Cells were harvested 24 h after transfection, washed three times with ice-cold PBS, and sonicated in TBS buffer (50 mM Tris-HCl, pH 8.0; 150 mM NaCl) using a Sonoplus Ultrasonic Homogenizer HD 3100 (Bandelin, MS72 probe) at 15% output power for 3 × 10 s. Homogenates were centrifuged at 1,000 × g for 10 min at 4°C to remove nuclei and unbroken cells. Protein concentration of the supernatant was determined using Bradford reagent (Bio-Rad, Hercules, CA) with BSA as a standard. Lysates were aliquoted and stored at −80°C until further use.

Solid-phase interaction assays were performed as described previously (32). Briefly, 96-well polystyrene plates (MaxiSorp, Nalgen Nunc Int., Rochester, NY, USA) were coated overnight at 4°C with 1.8 μg/well of purified 3xFLAG-PNPLA1, PLIN2, or PLIN3 in 100 μl of coating buffer (50 mM Tris-HCl, pH 8.0; 200 mM NaCl). The next day, wells were blocked for 3 h at room temperature with 200 μl of 5% (w/v) delipidated BSA (Sigma-Aldrich) in TBS buffer (50 mM Tris-HCl, pH 8.0; 150 mM NaCl). Subsequently, 40 μg of total protein from cell lysates (containing Xpress-tagged ABHD5 variants or β-Gal) diluted in 100 μl of dilution buffer (TBS buffer containing 2% BSA and 0.05% Tween 20) were added to each well in triplicates and incubated overnight at 4°C. Background controls (coated with buffer only) were included to determine non-specific binding. After incubation, wells were washed three times with 200 μl of TBS buffer containing 0.05% Tween 20 (Sigma-Aldrich). Plates were then incubated for 1 h at room temperature with anti-Xpress antibody (1:2,500; Thermo Fisher Scientific) diluted in wash buffer containing 0.5% delipidated BSA, followed by three washes and a 1 h incubation with HRP-conjugated anti-mouse IgG secondary antibody (1:2,500; NA931V; Cytiva) in the same buffer. Following final washes, antibody binding was visualized by adding 100 μl of tetramethylbenzidine (TMB) substrate solution (27 mM citric acid, 51 mM Na_2_HPO_4_, 0.1 μg/μl TMB, 2 mM H_2_O_2_) per well, and absorbance was measured at 620 nm. Background-subtracted values were used for quantification and statistical analysis. To account for differences in expression levels of ABHD5 variants, quantitative immunoblotting of cell lysates was performed using Image Lab Software (v6.0.1; Bio-Rad). Band intensities were normalized to GAPDH, and expression values were adjusted relative to wild-type ABHD5 levels.

### Confocal laser scanning microscopy

For live-cell imaging experiments, COS-7 cells (2 × 10^4^ per well) were seeded in ibiTreat 8-well chamber slides (ibidi GmbH, Gräfelfing, Germany) with a specialized polymer coverslip bottom. Cells were co-transfected with 100 ng each of pEYFP-PNPLA1 (p.S53L), pEYFP-PLIN2, or pEYFP-PLIN3 and 100 ng of pECFP-ABHD5 variants using Lipofectamine 3000 Reagent (Thermo Scientific Fisher), following the manufacturer’s instructions. Eight hours post-transfection, cells were incubated with 100 μM oleic acid complexed with delipidated BSA at a molar ratio of 3:1 to induce LD formation.

At 24 h post-transfection, neutral lipids were stained using HCS LipidTOX Deep Red Neutral Lipid Stain (1:5,000; Thermo Fisher Scientific) according to the manufacturer’s protocol. Imaging was performed on a Leica SP8 DLS confocal laser scanning microscope (Leica Microsystems, Wetzlar, Germany) equipped with a Leica PL APO HCX 63 × 1.4 numerical aperture oil immersion objective. Fluorophores were excited and detected using the following settings: Cyan-fluorescent protein (CFP) – excitation at 458 nm, emission between 465 - 490 nm; Yellow-fluorescent protein (YFP) – excitation at 514 nm, emission between 525 - 585 nm; LipidTOX Deep Red– excitation at 633 nm, emission between 650 – 750 nm. Detector and laser gain were adjusted to avoid signal saturation or photobleaching. Image analysis was performed using Fiji (ImageJ; NIH, Bethesda, MD).

### *In vitro* AcylCer synthesis assay

Proteoliposomes containing 3xFLAG-tagged PNPLA1, ABHD5 variants, or the negative control protein taste receptor type 1 member 1 (T1R1) were generated by *in vitro* translation using the wheat germ-based ProteoLiposome Expression Kit (CellFree Sciences), as previously described (17). For *in vitro* transcription, pEU-E01-plasmids encoding the respective open-reading frames were transcribed using SP6 RNA polymerase according to the manufacturer's instructions. Liposomes were prepared by drying a lipid mixture consisting of 5 mg phosphatidylcholine (PC; 1-palmitoyl-2-oleoyl-glycero-3-phosphocholine, Avanti Polar Lipids), 50 μg trilinolein (glyceryl trilinoleate; Sigma-Aldrich), and 50 μg ω-OH-ceramide (ceramide d18:1/30:0; Cayman Chemical) under a gentle nitrogen stream. The resulting lipid film was emulsified by incubation for 1 h in 250 μl of 1 × SUB-AMIX SGC at room temperature, followed by sonication (Sonoplus HD 3100; Bandelin, MS72 probe) for 3 × 30 s at 20% output power.

For co-expression of PNPLA1 with ABHD5 variants or T1R1 in liposomes, *in vitro* translation reactions were prepared by mixing: 5 μl liposomes, 6.25 μl WEPRO7240, 0.1 μl creatine kinase (20 mg/ml), 1.88 μl of *PNPLA1* mRNA, 1.88 μl *ABHD5* or *T1R1* mRNA, 6.15 μl 1 × SUB-AMIX SGC, and 3.74 μl of nuclease-free water. The final mixture was carefully transferred to the bottom of a 96-well plate containing 175 μl 1xSUB-AMIX SGC translation buffer and incubated for 20 h at 15°C to allow bilayer formation and protein synthesis.

Proteoliposomes were harvested by centrifugation at 20,000 × g for 10 min at 4°C and washed three times with ice-cold PBS. Pellets were resuspended in assay buffer (50 mM HEPES buffer, pH 7.4; 150 mM NaCl; 10% glycerol) by mild sonication (Sonoplus HD 3100, Bandelin, MS72 probe) for 2 × 10 s at 10% output power. Protein concentrations were determined using the Bradford assay (Bio-Rad). Expression of target proteins was confirmed by immunoblotting using 1 μg of total protein.

To assess PNPLA1-mediated acylCer synthesis activity of proteoliposomes, 4 μg of total protein were incubated for 30 min at 37°C in a water bath. Reactions were stopped by adding 1 ml MTBE/methanol (3/1, v/v) containing 500 nmol BHT, 0.1% acetic acid, and internal lipid standards. Lipids were extracted as described above and dried under a gentle nitrogen stream. Samples were reconstituted in 50 μl 2-propanol/methanol/ddH_2_O (70/25/10, v/v/v), and 5 μl were subjected to targeted UHPLC-MS/QQQ analysis.

### Immunoblotting

Equal amounts of proteins (as indicated in the figure legends) were separated by SDS-PAGE and transferred onto a PVDF membrane (pore size 0.45 μm; Carl Roth GmbH, Karlsruhe, Germany) according to standard protocols. Specific proteins were detected using the following antibodies: anti-Xpress® (1:5,000; R910-25; Thermo Fisher Scientific) with HRP-conjugated anti-mouse secondary antibody (1:10,000; NA931V; Cytiva), anti-6X His tag® (1:3,000; ab18184; Abcam) with the same secondary antibody, monoclonal HRP-conjugated anti-FLAG M2 (1:5,000; A8592; Sigma-Aldrich), or anti-GAPDH antibody (1:10,000; 2118S; Cell Signaling Technology, Danvers, MA) with HRP-conjugated anti-rabbit secondary antibody (1:10,000, 7074P2; Cell Signaling Technology). Specifically bound immunoglobulins were visualized using Clarity Western ECL substrate (Bio-Rad) and analyzed with the ChemiDoc Detection System (Bio-Rad). To verify equal protein loading, PVDF membranes were stained with Coomassie Blue staining solution (50% (v/v) ethanol, 8% (v/v) acetic acid, 0.25% (w/v) Coomassie Brilliant Blue R250), followed by destaining with 8% (v/v) acetic acid and 30% (v/v) methanol.

### Statistical analysis

Statistical analyses were performed using GraphPad Prism (v10.4.1.; GraphPad Software, San Diego, CA). Group differences were determined using one-way ANOVA with post-hoc comparisons against the control groups performed using Tukey’s test or, alternatively, by a two-tailed Student’s *t* test. Data are presented as means + SD. Statistical significance was defined as follows: *P* < 0.05 (∗, $), *P* < 0.01 (∗∗, $$), and *P* < 0.001 (∗∗∗, $$$).

## Results

### Mutants associated with ABHD5-sEDD fail to stimulate PNPLA1-mediated acylCer biosynthesis

ABHD5 and PNPLA1 are both essential for the final step in acylCer biosynthesis, where ω-OH-ceramides are esterified with LA (Fig. 1B). Previous work from our laboratory has shown that ABHD5 directly interacts with PNPLA1, stimulating its transacylase activity and thereby facilitating acylCer formation (18). However, the molecular mechanism by which ABHD5 activates PNPLA1 remains unclear. Given the importance of acylCers in maintaining skin barrier integrity, especially in the context of ichthyosis, we aimed to investigate how mutations in *ABHD5* affect the protein's ability to stimulate PNPLA1.

For this purpose, we selected seven clinically reported ABHD5 point mutants (Fig. 1A) based on their relevance and mutation type (Table 1), and co-expressed them with PNPLA1 and the essential enzymes required for the synthesis of ω-OH ceramides in HEK293T cells. Successful overexpression of all enzymes was confirmed by immunoblot analysis (Fig. 1C). To assess the functional consequences of the *ABHD5* mutations on PNPLA1-mediated acylCer biosynthesis, we then measured acylCer content in cells using targeted mass spectrometry.

**Table 1:** Clinical and biochemical phenotypes associated with pathogenic ABHD5 variants investigated in this study

ABHD5 Mutant	Skin Phenotype	Neutral lipid Storage Phenotype	Liver Phenotype	Other Symptoms	References
p.H82R	NCIE	Ectopic lipid accumulation including Jordans’ anomaly	Not reported	Muscle weakness, myalgia, hearing loss, cataract, elevated creatine kinase levels	(24)
p.S115G	NCIE	Ectopic lipid accumulation including Jordans’ anomaly	MASH	Splenomegaly, elevated levels of muscle enzymes	(23)
p.Q130P	NCIE	Ectopic lipid accumulation including Jordans’ anomaly	Not reported	Bilateral sensory deafness, mental retardation	(4)
p.H251P	NCIE	Ectopic lipid accumulation including Jordans’ anomaly	MASH	Elevated creatine kinase levels, muscle pain and weakness, growth retardation, mental retardation, deafness, kidney involvement, cataract	(25, 28)
p.E260K	NCIE with more severe erythematous phenotype	Ectopic lipid accumulation including Jordans’ anomaly	Hepatomegaly	Ectropion, eclabion, alopecia	(4)
p.G271R	NCIE with unusual dermatological findings: unaffected areas between the plaques on the back and the limbs; seborrheic dermatitis-like mild erythema and fine scales on face and scalp	Ectopic lipid accumulation including Jordans’ anomaly	SLD	Elevated creatine kinase levels	(26)
p.R297N	NCIE	Ectopic lipid accumulation including Jordans’ anomaly	Hepatomegaly	Limb weakness, lumbar lordosis, myopathic gait, myalgia, elevated creatine kinase levels	(27, 29, 30)

This table summarizes genotype-phenotype relationships reported in patients with specific *ABHD5* missense mutations. Documented clinical features include skin abnormalities, neutral lipid storage phenotypes, liver involvement, and other systemic manifestations. Phenotypic variability is reflected in the extent and severity of organ involvement across individual cases.

MASH, Metabolic dysfunction-associated steatohepatitis; NCIE, non-bullous congenital ichthyosiform erythroderma; SLD, steatotic liver disease.

Consistent with our previous study (18), we found that cells overexpressing wild-type ABHD5 exhibited approximately a 5-fold increase in acylCer content compared to cells transfected with the negative control β-galactosidase (β-Gal). This significant increase in acylCer formation clearly demonstrates the stimulating effect of wild-type ABHD5 on PNPLA1-mediated acylCer synthesis. In contrast, cells expressing the ABHD5 missense mutants showed significantly lower acylCer levels than those overexpressing wild-type ABHD5 (Fig. 1D), highlighting the essential role of ABHD5 for PNPLA1-mediated acylCer synthesis and suggesting that even single amino acid substitutions in ABHD5 can severely affect sphingolipid metabolism. Overall, our results clearly demonstrate that the ABHD5 mutants frequently associated with ABHD5-sEDD are unable to effectively stimulate PNPLA1, leading to decreased acylCer levels.

### Disease-associated ABHD5 mutants exhibit altered protein binding to PNPLA1

**Fig. 2:**
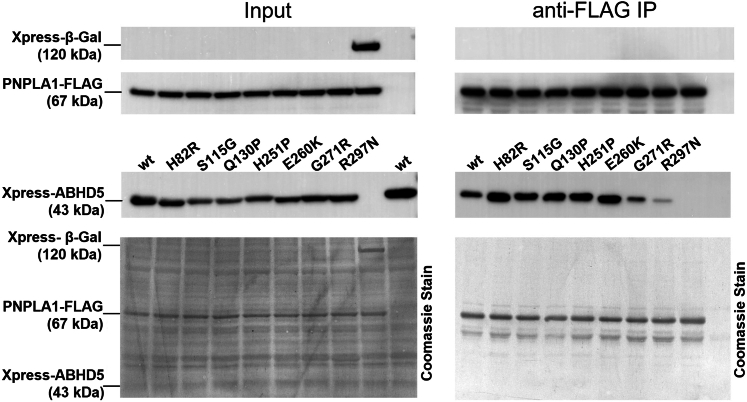
Increased or decreased binding affinities of disease-associated ABHD5 mutants for PNPLA1. Xpress-tagged human ABHD5 mutants co-immunoprecipitate with FLAG-tagged human PNPLA1. ABHD5 variants and β-galactosidase (β-Gal) were detected in HEK293T cell lysates (Input) and anti-FLAG immunoprecipitation (IP) fraction by immunoblot analysis using respective antibodies. Coomassie Blue staining indicates comparable protein loading.

To investigate whether the impaired acylCer formation of cells co-expressing PNPLA1 and ABHD5 mutants is linked to a reduced protein-protein interaction of these mutants with PNPLA1, we performed co-immunoprecipitation assays using cell lysates from HEK293T cells co-expressing FLAG-tagged PNPLA1 and Xpress-tagged ABHD5 variants or β-Gal as a negative control (Fig. 2). To ensure specificity of the protein interaction, Xpress-tagged wild-type ABHD5 was co-expressed with the FLAG empty vector as a negative control to exclude nonspecific binding of ABHD5 to the anti-FLAG-tagged antibody-coated agarose beads.

Consistent with our previous study (18), we observed that Xpress-tagged wild-type ABHD5 co-immunoprecipitated with FLAG-tagged PNPLA1, confirming a direct physical interaction between these two proteins. Importantly, the ABHD5 variants p.G271R and p.R297N showed somewhat decreased binding ability to PNPLA1 compared to the wild-type protein, as evident by lower band intensities in the immunoblot analysis (Fig. 2), suggesting that these specific point mutations impair the protein-protein interaction between ABHD5 and PNPLA1. Surprisingly, the other ABHD5 mutants exhibited rather increased binding affinities for PNPLA1 (increased band intensities) compared to the wild-type protein, highlighting the need for additional studies to assess how these amino acid substitutions in ABHD5 affect PNPLA1-mediated acylCer synthesis.

**Fig. 3:**
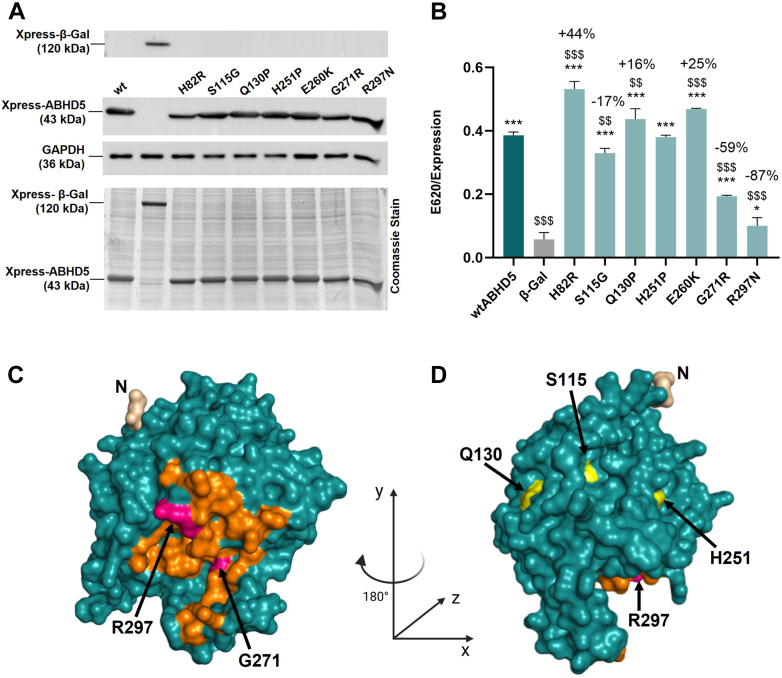
Determination of ABHD5 binding affinities for PNPLA1 using solid-phase interaction assays. A: Detection of Xpress-tagged human ABHD5 mutants and β-galactosidase (β-Gal) in HEK293T cell lysates for solid-phase interaction assays by immunoblot analysis using respective antibodies. Detection of GAPDH and Coomassie Blue staining indicate equal protein loading. B: Solid-phase interaction assays quantify the binding affinities of ABHD5 mutants for PNPLA1. Data are presented as means of triplicates + SD and are representative of three independent experiments. Statistical differences were determined using one-way ANOVA (∗, *P* < 0.05; ∗∗, *P* < 0.01; ∗∗∗, *P* < 0.001; compared to the negative control β-Gal, and $, *P* < 0.05; $$, *P* < 0.01; $$$, *P* < 0.001; compared to wtABHD5). C, D: 3D models of ABHD5 generated using AlphaFold and visualized in PyMOL. The N-terminus, labeled “N” and colored in wheat in both panels, is shown at its native position based on the predicted topology and secondary structure alignment of the model. Panel C shows the AlphaFold-predicted structure in its original orientation, with the predicted ABHD5-PNPLA1 interaction interface highlighted in orange. Key residues G271 and R297 within this interface are depicted in magenta. A black, curved arrow indicates the ∼180° rotation around the y-axis used to generate the alternative view shown in Panel D. This rotated perspective exposes the residues S115, Q130, and H251, shown in yellow, which are positioned opposite the PNPLA1 binding surface. Although residues H82 and E260 are functionally relevant, they are not surface-exposed in this structural model and are therefore not depicted in this orientation.

To re-evaluate the results of the ABHD5-PNPLA1 co-immunoprecipitation experiments and to employ a more quantitative assessment of the ABHD5-PNPLA1 protein interaction, we performed solid-phase interaction assays. For this purpose, full-length human PNPLA1 was partially purified from Expi293F cell lysates using ÄKTA pure™ affinity chromatography (Supplemental Fig. S1A) and subsequently used to coat 96-well plates. Then, lysates from HEK293T cells expressing Xpress-tagged ABHD5 mutants or β-Gal as a negative control were added, and the plates were incubated overnight. Bound ABHD5 was then detected using respective primary and HRP-labeled secondary antibodies and the chromogenic substrate TMB. The absorbance at 620 nm was measured and normalized to the protein levels of wild-type ABHD5 and mutants or β-Gal in the lysates as determined by immunoblot analysis (Fig. 3A). The normalized absorbance was directly proportional to the binding affinity of each ABHD5 variant for PNPLA1.

Consistent with our co-immunoprecipitation results, we observed that the disease-associated mutants p.G271R (−59%) and p.R297N (−87%) displayed drastically reduced PNPLA1 binding affinities compared to wild-type ABHD5 (Fig. 3B). These findings align with our predicted 3D model (33) of ABHD5 (UniProt; Q8WTS1), which suggests that amino acid residues G271 and R297 reside on the protein surface in regions critical for protein-protein interactions. These ABHD5 residues are located close to the putative interaction interface with PNPLA1 (Fig. 3C), attributing these residues a crucial role in mediating the ABHD5-PNPLA1 interaction. In contrast, all other ABHD5 mutants exhibited either significantly increased (p.H82R, +44%; p.Q130P, +16%; and p.E260K, +25%), somewhat reduced (p.S115G, −17%), or unaffected binding (p.H251P) to PNPLA1 compared to wild-type ABHD5 (Fig. 3B). Upon mapping these essential amino acid residues onto our ABHD5 3D structure, we found that they are located on the opposite side of the predicted PNPLA1 interaction surface on the protein (Fig. 3D). Based on these findings, we conclude that these residues are unlikely to directly interfere with PNPLA1 binding but may play a role in other processes such as protein folding, stability, allosteric modulation, or the intracellular localization of ABHD5. Taken together, these results indicate that impaired protein-protein interactions between ABHD5-sEDD-associated mutants and PNPLA1 only partially account for reduced acylCer biosynthesis in patients, implying that additional mechanisms likely contribute to the pathogenesis of the disease.

### PNPLA1 fails to localize to intracellular LDs in the presence of disease-associated ABHD5 mutants

In our previous study (18), we demonstrated that ABHD5 localizes to intracellular LDs and is essential for recruiting PNPLA1 to these organelles. To investigate whether ABHD5-sEDD-associated mutants disrupt this process, we performed live-cell imaging experiments with COS-7 cells co-expressing CFP-tagged ABHD5 variants and a catalytically inactive YFP-tagged PNPLA1 mutant (p.S53L, YFP-PNPLA1^∗^) (34). The YFP-PNPLA1^∗^ mutant was used to prevent the enzymatic activity of PNPLA1 (35), thereby facilitating undisrupted LD formation in transfected COS-7 cells and allowing us to study the recruitment and localization of PNPLA1. LDs were visualized using HCS LipidTOX™ Deep Red Neutral Lipid Stain.

**Fig. 4:**
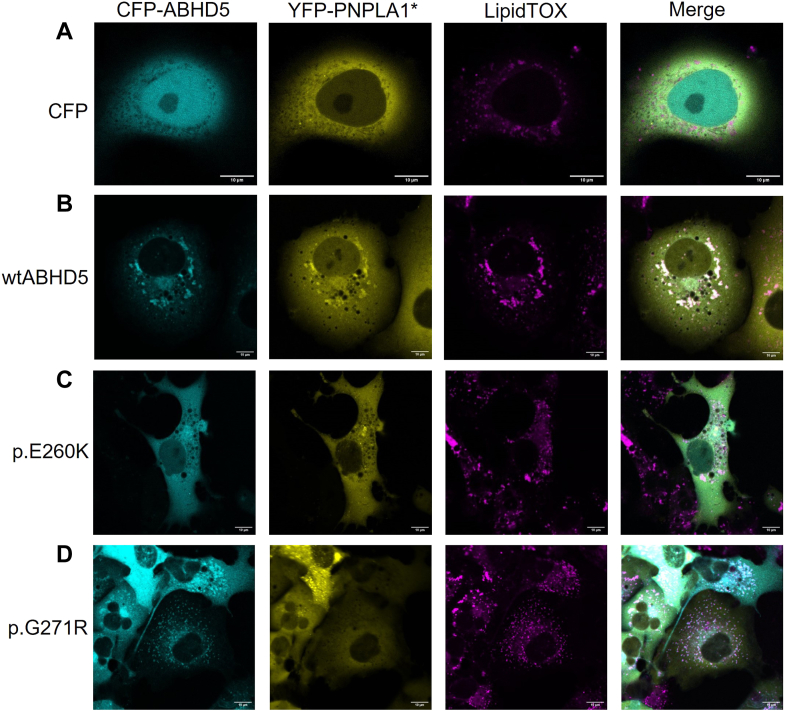
PNPLA1 fails to localize on intracellular LDs in the presence of disease-associated ABHD5 mutants. The co-localization of fluorescent-labeled proteins was analyzed by confocal laser scanning microscopy in transfected COS-7 cells incubated with oleic acid to induce lipid droplet (LD) formation. Neutral lipids were stained using LipidTOX™ Deep Red Neutral Lipid Stain. A: Cells co-expressing CFP and a catalytically inactive YFP-tagged PNPLA1 mutant (p.S53L, YFP-PNPLA1^∗^) show cytosolic distribution of CFP and YFP signals. B: Cells co-expressing wild-type CFP-ABHD5 and YFP-PNPLA1^∗^ show co-localization of CFP and YFP signals at intracellular LDs. C: Cells co-expressing CFP-ABHD5 mutant p.E260K and YFP-PNPLA1^∗^ exhibit predominant cytosolic distribution of both YFP and CFP signals, with no clear co-localization at LDs. D: Cells co-expressing CFP-ABHD5 mutant p.G271R and YFP-PNPLA1^∗^ show CFP localization to intracellular LDs and YFP signals primarily in the cytosol and to structures close to the nucleus, indicating impaired PNPLA1 recruitment to LDs. Representative images are shown. Scale bars represent 10 μm.

As expected, and in line with our previous study (18), fluorescence microscopy of COS-7 cells co-transfected with YFP-tagged PNPLA1^∗^ and the empty pECFP-C1 vector revealed a primarily cytosolic distribution of YFP-PNPLA1^∗^ with additional localization near the nucleus, suggesting that PNPLA1 does not inherently localize to intracellular LDs (Fig. 4A). However, upon co-expression with CFP-tagged wild-type ABHD5, we observed a striking redistribution of YFP-PNPLA1^∗^ to intracellular LDs, where it strongly co-localized with ABHD5 (Fig. 4B). These findings clearly demonstrate the critical role of ABHD5 in targeting PNPLA1 to LDs, thereby ensuring access to its substrate and enabling efficient enzymatic activity.

Building on these results, we next addressed whether the proper LD targeting of PNPLA1 also depends on the correct intracellular localization of ABHD5. Consistent with this hypothesis, we found that the ABHD5-sEDD-associated mutants p.H82R, p.S115G, p.Q130P, p.H251P, and p.E260K, which exhibited similar or significantly increased binding affinities for PNPLA1 in our solid-phase interaction assays (Fig. 3B), failed to localize to LDs and instead showed predominantly cytosolic distribution in live-cell imaging experiments (Fig. 4C and Supplemental Fig. S2A–D). Consequently, PNPLA1 co-localized with these mutants in the cytosol, as shown in the merged images, indicating minimal PNPLA1 association with LDs in the presence of these ABHD5 variants.

In contrast, the ABHD5 mutants p.G271R and p.R297N localized to intracellular LDs (Fig. 4D and Supplemental Fig. S2E) but failed to recruit PNPLA1 to its substrate due to their significantly reduced binding affinities for PNPLA1, as shown in our co-immunoprecipitation and solid-phase interaction assays (see also Fig. 3B). Consequently, PNPLA1 did not co-localize with these ABHD5 mutants at LDs but instead showed localization to structures near the nucleus, indicating a shift in intracellular distribution. Taken together, these findings provide compelling evidence that the presence of functional ABHD5 is critical for the correct localization of PNPLA1 to LDs to facilitate access to its substrate. Disease-associated amino acid substitutions in ABHD5 disrupt either its interaction with PNPLA1 or its localization to LDs, both of which ultimately impair PNPLA1 recruitment/localization and its enzymatic function, thereby leading to the pathogenesis of ichthyosis.

### Point mutations associated with ABHD5-sEDD affect the binding affinities of ABHD5 for PLIN2 or PLIN3

**Fig. 5:**
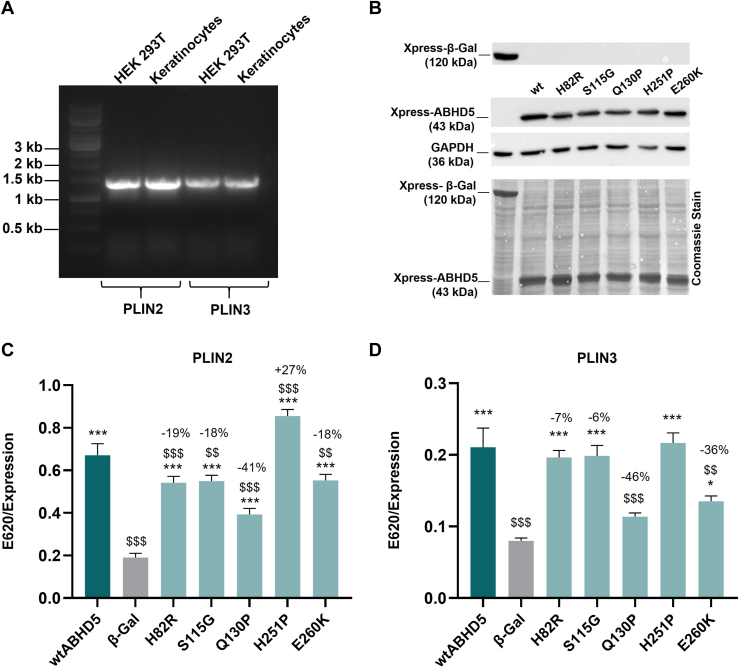
Disease-associated ABHD5 mutants show impaired binding affinities for PLIN2 and PLIN3. A: Reverse transcription PCR analysis of *PLIN2* and *PLIN3* mRNA expression in HEK293T cells and terminally differentiated human primary keratinocytes. B: Immunoblot analysis of Xpress-tagged human ABHD5 variants and β-galactosidase (β-Gal) in HEK293T cell lysates used for solid-phase interaction assays. Respective antibodies were used for detection. Coomassie Blue staining shows equal protein loading. C: Analysis of ABHD5 binding affinities for PLIN2 using solid-phase interaction assays. D: Analysis of ABHD5 binding affinities for PLIN3 using solid-phase interaction assays. Data are presented as means of triplicates + SD and are representative of three independent experiments. Statistically significant differences were determined by one-way ANOVA (∗, *P* < 0.05; ∗∗, *P* < 0.01; ∗∗∗, *P* < 0.001 compared to the negative control β-Gal and $, *P* < 0.05; $$, *P* < 0.01; $$$, *P* < 0.001 compared to wtABHD5).

Given the observed mislocalization of the ABHD5 mutants p.H82R, p.S115G, p.Q130P, p.H251P, and p.E260K, we next investigated whether the impaired LD targeting of PNPLA1 in the presence of these ABHD5 mutants is linked to altered binding affinities for key LD-coating proteins, such as PLIN2 and PLIN3. In non-adipose and non-oxidative tissues, PLIN2 and PLIN3 regulate lipid metabolism like PLIN1 and PLIN5 in adipose and muscle tissue, respectively, by controlling lipase access to LDs and modulating their activity by releasing ABHD5 from PLIN proteins for interaction with lipases (21). Based on this fact and consistent with data from The Human Protein Atlas (data available from v24.proteinatlas.org) (36), reverse transcription PCR analysis detected robust mRNA expression of *PLIN2* and *PLIN3* in both HEK293T cells and terminally differentiated human primary keratinocytes (Fig. 5A).

To assess the binding between the ABHD5 variants and these perilipins, we again performed solid-phase interaction assays using partially purified PLIN2 or PLIN3 from Expi293F cell lysates (Supplemental Fig. S1B, C) and lysates from HEK293T cells overexpressing the different ABHD5 mutants or β-Gal as a negative control (Fig. 5B). As illustrated in Figure 5C, D, our interaction analysis of the ABHD5-sEDD mutants p.H82R, p.S115G, p.Q130P, and p.E260K revealed significantly reduced binding affinities for PLIN2 and PLIN3 compared to wild-type ABHD5. Interestingly, only the p.H251P mutant showed unaffected or significantly increased binding to PLIN2 and PLIN3, respectively, suggesting that this specific mutation alters the functional properties of the protein, enhancing its interaction with these LD-coating proteins *in vitro* (Fig. 5C, D). Overall, our findings suggest that impaired binding of mutant ABHD5 to PLIN2 or PLIN3 may consequently affect proper PNPLA1 localization to intracellular LDs, thereby contributing to defective PNPLA1-mediated acylCer synthesis in ABHD5-sEDD.

### Point mutations associated with ABHD5-sEDD affect the co-localization of ABHD5 with PLIN2 and PLIN3 at intracellular LDs

To further validate the results of the ABHD5-PLIN2/PLIN3 solid-phase interaction assays *in vivo*, we performed live-cell imaging of COS-7 cells co-expressing CFP-tagged ABHD5 variants with YFP-tagged PLIN2 or PLIN3. To induce LD formation, cells were loaded with oleic acid, and LDs were visualized using HCS LipidTOX™ Deep Red Neutral Lipid Stain.

**Fig. 6:**
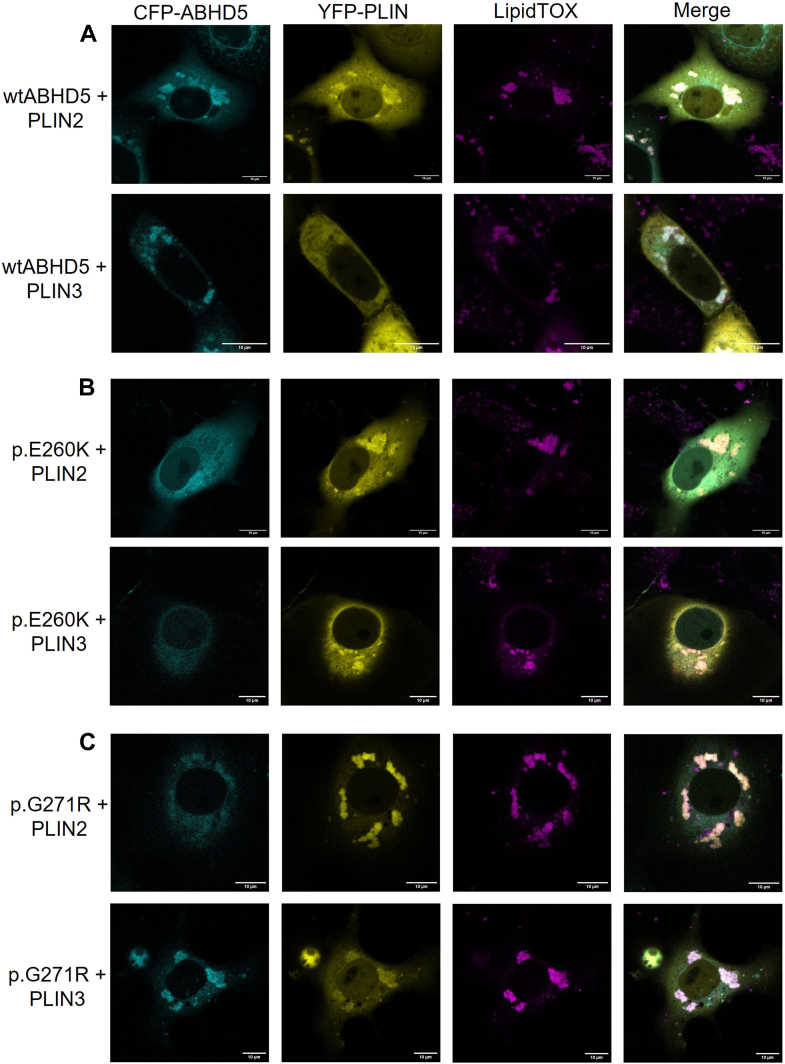
Intracellular localization of disease-associated ABHD5 mutants in the presence of PLIN2 or PLIN3. The subcellular distribution of fluorescently labeled proteins was analyzed by confocal-laser scanning microscopy in transfected COS-7 cells incubated with oleic acid to induce lipid droplet (LD) formation. Neutral lipids were visualized with HCS LipidTOX™ Deep Red Neutral Lipid Stain. A: Cells co-expressing wild-type CFP-ABHD5 and YFP-PLIN2 or PLIN3 show strong co-localization at intracellular LDs, indicated by increased CFP and YFP signal. B: Cells expressing the CFP-ABHD5 variants p.E260K together with YFP-PLIN2 or PLIN3 show a mislocalization of CFP-ABHD5, with an increased CFP signal in the cytosol, while YFP-PLIN2 or PLIN3 remain enriched at LDs. C: The CFP-ABHD5 variant p.G271R co-expressed with YFP-PLIN2 or PLIN3 still localize to LDs, showing both CFP and YFP signal at intracellular LDs. Representative images are shown. Scale bars represent 10 μm.

PLIN2 and PLIN3 are well-established interaction partners of ABHD5, playing key roles in lipid metabolism (16, 21). As expected, CFP-tagged wild-type ABHD5 co-localized with both YFP-tagged PLIN2 and PLIN3 (Fig. 6A) at the surface of intracellular LDs, suggesting a direct protein-protein interaction. In contrast, the disease-associated ABHD5 mutants p.H82R, p.S115G, p.Q130P, p.H251P, and p.E260K failed to co-localize with PLIN2 or PLIN3 to LDs, as indicated by an evenly distributed CFP signal, consistent with their predominant cytosolic distribution (Fig. 6B and Supplemental Figs. S3A–D and S4A–D). Interestingly, the ABHD5-sEDD-associated mutants p.G271R and p.R297N exhibited a markedly different localization pattern (Fig. 6C and Supplemental Figs. S3E and S4E). The CFP signals of these mutants overlapped with the YFP signal of PLIN2 or PLIN3, as well as with the LipidTOX signal, demonstrating their co-localization with PLIN proteins at the LD surface.

Taken together, these findings demonstrate that the ABHD5-sEDD-associated mutants p.H82R, p.S115G, p.Q130P, p.H251P, and p.E260K show impaired LD targeting, which is likely at least partially linked to their reduced binding affinities for PLIN2 and PLIN3. In contrast, point-mutations located within the ABHD5-PNPLA1 interaction surface, ie, p.G271R and p.R297N, do not disrupt the ABHD5 localization to LDs, again demonstrating the functional differences among disease-associated mutants.

### Disease-associated ABHD5 mutants and PNPLA1 co-expressed on artificial liposomes stimulate acylCer synthesis

**Fig. 7:**
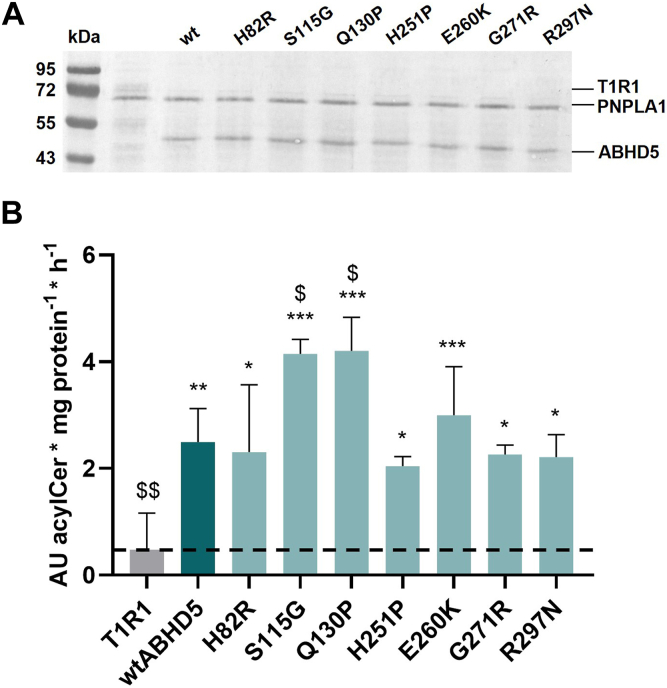
Co-expression through *in vitro* translation of human PNPLA1 and disease-associated ABHD5 point mutants in proteoliposomes restores full acylCer synthesis activity of PNPLA1. A: Expression analysis indicates similar protein amounts of PNPLA1 and ABHD5 variants in purified proteoliposomes using Coomassie Blue staining. B: PNPLA1-mediated acylCer synthesis activity of proteoliposomes in the presence of specific ABHD5 mutants. AcylCer levels were determined using UHPLC- MS/QQQ. The dashed line indicates basal PNPLA1-mediated acylCer synthesis activity. Data are presented as means of quadruplicates + SD and are representative of three independent experiments. Statistically significant differences were determined using one-way ANOVA (∗, *P* < 0.05; ∗∗, *P* < 0.01; ∗∗∗, *P* < 0.001; compared to the control T1R1, and $, *P* < 0.05; $$, *P* < 0.01; compared to wtABHD5).

To further assess the functional consequences of ABHD5 mislocalization on PNPLA1-mediated acylCer synthesis, we investigated whether ABHD5-sEDD-associated mutants retain the ability to co-active PNPLA1 transacylase activity when artificially co-localized with PNPLA1 on LDs. For this purpose, we used a cell-free *in vitro* translation system to co-reconstitute PNPLA1 and ABHD5 mutants into artificial liposomes containing trilinolein and ω-OH-ceramide as substrates. This approach enforces membrane co-localization and allows the analysis of acylCer synthesis as a direct measure of PNPLA1 transacylase activity. All seven disease-associated ABHD5 mutants were analyzed together with wild-type ABHD5 in this assay. As a negative control, we replaced ABHD5 with the taste receptor type 1 member 1 (T1R1), which does not modulate PNPLA1 enzyme activity (Supplemental Fig. S5). This assay setup allowed us to analyze the ability of each ABHD5 variant to stimulate PNPLA1 activity, independent of its subcellular localization. Expression analysis confirmed similar protein levels of PNPLA1 and ABHD5 variants in the purified proteoliposomes with an approximate ABHD5-to-PNPLA1 ratio of 1:1 (Fig. 7A). The proteoliposomes were then incubated at 37°C, and the formation of acylCers was quantified by mass spectrometry to determine PNPLA1 enzyme activity.

As shown in Figure 7B, we detected low basal PNPLA1 activity in the presence of the negative control T1R1. Co-expression of PNPLA1 with wild-type ABHD5 increased acylCer synthesis by approximately 5-fold compared to basal conditions, clearly demonstrating the stimulatory effect of ABHD5 on PNPLA1 activity under these experimental conditions. Notably, when we expressed the ABHD5-sEDD-associated mutants p.H82R, p.S115G, Q130P, H251P, or p.E260K, which exhibited impaired LD targeting but retained a strong interaction with PNPLA1 in our experiments, PNPLA1 synthesized acylCers at levels comparable or higher to those measured with wild-type ABHD5. This observation indicates that restoring the localization of these ABHD5 mutants through forced *in vitro* translation into proteoliposomes is sufficient to maintain PNPLA1 co-activation.

Interestingly, when we analyzed the ABHD5 mutants p.G271R and p.R297N, which showed significantly reduced binding affinities for PNPLA1 in our assays, we found that PNPLA1-mediated acylCer formation was not significantly different between wild-type ABHD5 and these mutants when co-localized with PNPLA1 in proteoliposomes (Fig. 7B). This surprising finding suggests that spatial proximity between ABHD5 and PNPLA1 within proteoliposomes, rather than direct binding, is sufficient for full enzymatic co-activation of PNPLA1, revealing that ABHD5 is required for the intracellular localization of PNPLA1 to LDs, but its enzymatic co-activation does not require a direct protein-protein interaction.

## Discussion

In ABHD5-sEDD, disease-associated mutations not only cause a severe defect in skin barrier function but are also associated with ectopic TAG accumulation in various cells and tissues, including leukocytes – a hallmark known as Jordans’ anomaly (Table 1) (1, 2, 3, 6). This neutral lipid storage phenotype primarily results from impaired ABHD5-mediated activation of ATGL, the rate-limiting enzyme in intracellular lipolysis (37). While this finding established ABHD5 as a crucial regulator of lipolysis, it did not, however, explain the onset of ichthyosis in ABHD5-sEDD (1, 2, 4, 6). The role of ABHD5 in epidermal lipid metabolism first became evident by the observation that individuals with ATGL deficiency, unlike those lacking functional ABHD5, do not exhibit ichthyosis, strongly suggesting that ABHD5 exerts an ATGL-independent function in the epidermis (38). Consistent with this hypothesis, recent studies have demonstrated that the severe skin permeability defect in ABHD5-sEDD patients arises from defective acylCer production by PNPLA1, a close homologue of ATGL predominantly expressed in the epidermis (39). PNPLA1 catalyzes the transesterification of ω-OH-ceramides with linoleic acid to generate acylCers, which are essential components for maintaining skin integrity and barrier function (13, 14, 15, 17). While previous research form our group and others (18, 19) has established that PNPLA1, like ATGL, requires ABHD5 as a co-activator for full enzymatic activity, the precise molecular mechanisms leading to reduced acylCer formation in ABHD5-sEDD remained unclear.

### *ABHD5* mutations disrupt PNPLA1 localization and function

In this study, we identified a novel molecular mechanism driving the pathogenesis of ichthyosis in ABHD5-sEDD, shedding light on the complex interplay between epidermal lipid metabolism and skin barrier function. We demonstrated that ABHD5-sEDD-associated mutations disrupt either ABHD5-PNPLA1 binding or ABHD5 subcellular localization to LDs, thereby impairing PNPLA1 enzymatic function and ultimately leading to epidermal barrier dysfunction. Our findings revealed that ABHD5 mutants p.H82R, p.S115G, p.Q130P, p.H251P, and p.E260K failed to localize to LDs, instead displaying a predominant cytosolic distribution. This cellular mislocalization partially correlates with significantly reduced binding affinities for PLIN2 and PLIN3, two major LD-coating proteins in non-adipose tissues (21). Importantly, these observations support the hypothesis that ABHD5 relies on perilipins for LD association, a mechanism well established in adipocytes, where PLIN1 recruits ABHD5 to LDs and regulates its availability for ATGL-mediated lipolysis (22, 40, 41). In keratinocytes, however, PLIN2 and PLIN3 functionally replace PLIN1, arguing for a tissue-specific mechanism of PNPLA protein recruitment by ABHD5. Despite their expression in the epidermis, mutations in either *PLIN2* or *PLIN3* have not been linked to Mendelian skin disorder in humans so far. Furthermore, knockout mouse models of *Plin2* and *Plin3* do not exhibit obvious skin phenotypes (42, 43, 44, 45, 46, 47), suggesting a possible functional redundancy between the two proteins in the epidermis. This redundancy may mask the impact of single perilipin deficiency under physiological conditions but becomes relevant in the context of *ABHD5* mutations, which disrupt perilipin-mediated recruitment mechanisms.

Consistent with this hypothesis, our results demonstrate that impaired PLIN2/PLIN3-ABHD5 co-localization has clear functional consequences in keratinocytes. Although the cytosolically distributed ABHD5 mutants displayed normal or even increased binding affinities for PNPLA1, they failed to recruit PNPLA1 to LDs. As a result, these mutants were unable to enhance PNPLA1-mediated acylCer synthesis, ultimately impairing skin integrity and barrier formation. This observation aligns with data indicating that ABHD5 binding to PLIN2/PLIN3 plays a key role in regulating lipid metabolism in non-adipose and non-oxidative tissues (48). Interestingly, the p.H251P mutant retained strong binding affinities for PLIN2/PLIN3 *in vitro* yet still showed cytosolic distribution and altered PNPLA1 localization *in vivo*. Similarly, the p.H82R and p.S115G mutants also displayed cytosolic distribution despite only modest reductions in PLIN2/PLIN3 binding. These results suggest that, in addition to perilipin interaction, other structural factors contribute to ABHD5 function in LD targeting (30, 49, 50). One plausible mechanism involves a direct interaction between ABHD5 and the limiting membrane of LDs, which appears to be essential for stable recruitment (50). Pathogenic amino acid substitutions potentially interfere with this membrane interaction by inducing conformational changes in ABHD5, which in turn prevents its correct recruitment to LDs, even when perilipin binding is preserved *in vitro*. Moreover, post-translational modifications, such as phosphorylation by protein kinase A, have been shown to regulate ABHD5 subcellular localization and interaction with PLIN1 in adipose tissue (51), and may likewise affect LD membrane association or binding to PLIN2/3 in keratinocytes. Although we did not directly assess ABHD5 phosphorylation, it is plausible that certain missense mutations indirectly affect membrane targeting or PLIN binding by altering post-translational modification sites or patterns. Taken together, these structural and regulatory alterations may underlie the cytosolic mislocalization of ABHD5 mutants, thereby impairing PNPLA1 targeting to LDs and ultimately disrupting acylCer synthesis in ABHD5-sEDD.

Conversely, the ABHD5 mutants p.G271R and p.R297N maintained normal LD localization comparable to wild-type ABHD5 but exhibited drastically reduced binding affinities for PNPLA1. These findings are consistent with our predicted 3D structure of ABHD5, which suggests that these residues are part of the ABHD5-PNPLA1 interaction surface. Disease-associated mutations impairing LD binding were instead mapped on the opposite side or inner region of ABHD5, supporting the idea that the protein harbors at least two independent binding sites: one for PNPLA1 and another for perilipins/LDs. This dual functionality ensures the correct intracellular localization of both PNPLA1 and ABHD5 at intracellular LDs, representing a crucial step for efficient acylCer biosynthesis.

### PNPLA1 co-activation by ABHD5 follows a spatial rather than direct interaction model

A central question in lipid metabolism is how ABHD5 co-activates PNPLA proteins. While previous studies proposed a mechanism based on direct protein-protein interaction (52, 53), our findings on PNPLA1 and disease-associated ABHD5 mutants challenge this view and instead support a dual-function model of ABHD5 action. Using a cell-free *in vitro* system that enforces the co-localization of ABHD5 with PNPLA1 on substrate-containing liposomes, we demonstrated that the mislocalized ABHD5 mutants p.H82R, p.S115G, p.Q130P, p.H251P, and p.E260K supported elevated PNPLA1-mediated acylCer synthesis when artificially targeted to the same membrane compartment. This finding indicates that these variants retain their co-activating capacity despite their cytosolic distribution in living cells.

While correct localization of PNPLA1 by ABHD5 is a prerequisite, it alone does not suffice to ensure full enzymatic activity. Efficient acylCer synthesis additionally requires ABHD5 to function as a co-activator at the LD membrane. Supporting this conclusion, the ABHD5 mutants p.G271R and p.R297N, which exhibited markedly reduced PNPLA1 binding, likewise restored acylCer synthesis *in vitro* when correctly co-localized with PNPLA1. This observation suggests that the co-activating function of ABHD5 does not depend on a direct physical interaction with PNPLA1, but rather involves a distinct, yet unidentified mechanism. Potential modes of action include substrate presentation or channeling, modulation of the membrane microenvironment, enhanced removal of the reaction product, or even proteolytic processing of PNPLA1 by ABHD5 (50, 54). Together, our findings support a dual-function model in which ABHD5 is indispensable for PNPLA1 activity *in vivo* by ensuring both correct membrane targeting and enzymatic co-activation at the LD surface. However, the precise molecular mechanism underlying this enzyme co-activation remains unclear not only for PNPLA1 – but also for other members of the PNPLA family – and will require further investigations, including structural and functional studies to be fully understood.

### Genotypic and phenotypic variability in ABHD5-sEDD

The functional differences observed among ABHD5 mutants in our study align with the broader genetic and phenotypic heterogeneity in ABHD5-sEDD (55). To date, over 140 cases of ABHD5-sEDD have been reported, with 78 different mutations identified in the *ABHD5* gene (55). These mutations range from common genetic variations, including deletions, insertions, missense, nonsense, and splice-site mutations, to more unusual alterations such as large genomic deletions or the insertion of a transposable LINE-1 element within an intron, leading to aberrant *ABHD5* mRNA splicing. While all seven *ABHD5* missense mutations analyzed in this study resulted in a loss of function consistently associated with ichthyosis (specifically non-bullous congenital ichthyosiform erythroderma, NCIE) and ectopic lipid accumulation including Jordans’ anomaly, clinical manifestations such as hepatomegaly, neurological impairments, and myopathy varied among patients (Table 1), suggesting that mutation-specific effects on ABHD5 function contribute to the phenotypic heterogeneity observed in ABHD5-sEDD (4, 23, 24, 25, 26, 27). Recent studies indicate that ABHD5 interacts with multiple PNPLA enzymes (18, 56, 57) and LD-associated proteins (22, 32, 40, 48, 58, 59, 60, 61, 62) across different tissues, supporting the notion that distinct binding sites and affinities for its interaction partners may drive tissue-specific outcomes. For example, in the liver, PNPLA3 competes with ATGL for ABHD5 binding (56). The common PNPLA3 variant p.I148M, a major risk factor for metabolic dysfunction-associated steatotic liver disease (MASLD), exhibits increased ABHD5 binding affinity, thereby sequestering the co-factor from ATGL and impairing TAG degradation (56, 63). It is therefore likely that disease-associated *ABHD5* mutations not only disrupt interactions with PNPLA1 and ATGL (PNPLA2) but also alter binding dynamics with other metabolic regulators, potentially contributing to the genetic and clinical heterogeneity of ABHD5-sEDD.

## Therapeutic Implications and Future Directions

**Fig. 8:**
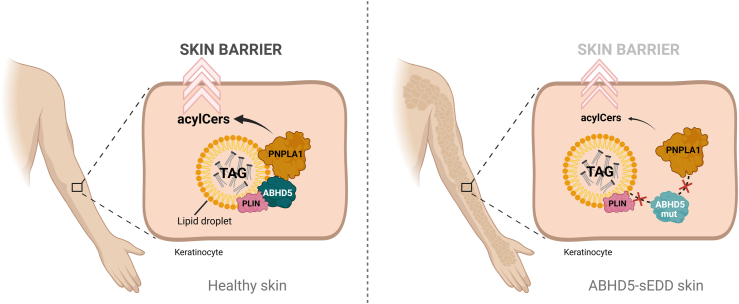
Proposed model of ABHD5–PNPLA1-mediated acylceramide biosynthesis in healthy skin and its disruption in ABHD5-syndromic epidermal differentiation disorder (ABHD5-sEDD). In healthy keratinocytes, ABHD5 functions as a critical co-activator of PNPLA1 by facilitating its co-localization with lipid droplets (LDs) through interactions with perilipins (PLIN). This spatial proximity enhances PNPLA1 access to lipid substrates and stimulates its transacylase activity, enabling efficient synthesis of ω-*O*-acylceramides (acylCers)–—sphingolipids essential for establishing the skin permeability barrier. In ABHD5-sEDD, pathogenic mutations in *ABHD5* impair either the association with perilipins/LDs or its interaction with PNPLA1, leading to reduced PNPLA1-LD localization, defective acylCer biosynthesis, and a compromised skin barrier, which underlies the ichthyosis phenotype observed in patients (created using BioRender.com).

Our findings provide critical insights into the molecular basis of ichthyosis pathogenesis in ABHD5-sEDD (Fig. 8). In healthy skin, our data demonstrate that ABHD5 functions as a key co-activator of PNPLA1 in keratinocytes by facilitating its co-localization with LDs. This process ensures that PNPLA1 is positioned close to its lipid substrates, thereby stimulating its transacylase activity and promoting the efficient synthesis of acylCers for maintaining skin permeability barrier function. In ABHD5-sEDD, pathogenic mutations disrupt this process by impairing either ABHD5-LD association or PNPLA1 interaction, resulting in defective acylCer biosynthesis and a compromised skin permeability barrier. Importantly, our data support a co-localization-driven model, in which ABHD5 directs PNPLA1 to LDs for efficient acylCer synthesis. Consistent with this model, spatial proximity of the proteins appears to be sufficient for proper PNPLA1 function, raising the exiting possibility that restoring the LD localization of both ABHD5 and PNPLA1 could serve as a viable therapeutic approach for ABHD5-sEDD. Targeting ABHD5-perilipin interactions or developing small molecules that promote PNPLA1 co-localization with LDs may provide novel strategies for therapeutic interventions (64).

In summary, our study identifies a previously unrecognized molecular mechanism for PNPLA1 enzyme function and highlights the critical role of ABHD5-mediated PNPLA1 localization to LDs. These findings not only enhance our understanding of epidermal lipid metabolism and skin barrier formation but also provide a new basis for the development of innovative therapeutic strategies for ABHD5-sEDD. Future research will focus on elucidating structural determinants of ABHD5-LD association and exploring potential pharmacological interventions aimed at restoring PNPLA1-LD localization in this disorder. Ultimately, these insights may support the design of targeted interventions to repair skin barrier abnormalities in affected individuals.

## Data availability

The data supporting the findings of this study are presented within the article and its supplementary materials. Additional information or raw data are available from the corresponding author upon reasonable request. Requests for materials should be addressed to Dr. Franz Radner (franz.radner@uni-graz.at), Institute of Molecular Biosciences, University of Graz, Heinrichstraße 31/1, 8010 Graz, Austria.

## Supplemental data

This article contains supplemental data.

## Conflict of interest

The authors declare that they have no conflicts of interest with the contents of this article.
