# Supplementary material for: Defective targeting of PNPLA1 to lipid droplets causes ichthyosis in ABHD5-syndromic epidermal differentiation disorder
Source: J Lipid Res. 2025 Aug 14;66(9):100875. doi: 10.1016/j.jlr.2025.100875 (PMC12465037; doi:10.1016/j.jlr.2025.100875)
Supplement: Supplemental Figures and Tables [file mmc1.docx]

**Supplemental Information:**

**Defective targeting of PNPLA1 to lipid droplets causes ichthyosis in ABHD5-syndromic epidermal differentiation disorder**

Margarita Schratter^1^, David Holubek^1^, Lukas Koeffler^1^, Thomas Züllig^1^, Thomas O. Eichmann^2,3^, Heimo Wolinski^1,4^, Monika Oberer^1,3,4^, Achim Lass^1,3,4^, and Franz P. W. Radner^1,3,4^

^1^ Institute of Molecular Biosciences, University of Graz, Graz, Austria.

^2^ Core Facility Mass Spectrometry, ZMF, Medical University of Graz, Graz, Austria.

^3^ BioTechMed-Graz, Graz, Austria.

^4^ Field of Excellence BioHealth, University of Graz, Graz, Austria.

**Supplemental Table 1: Primer sequences used for molecular cloning.** Restriction enzyme recognition sites are underlined.


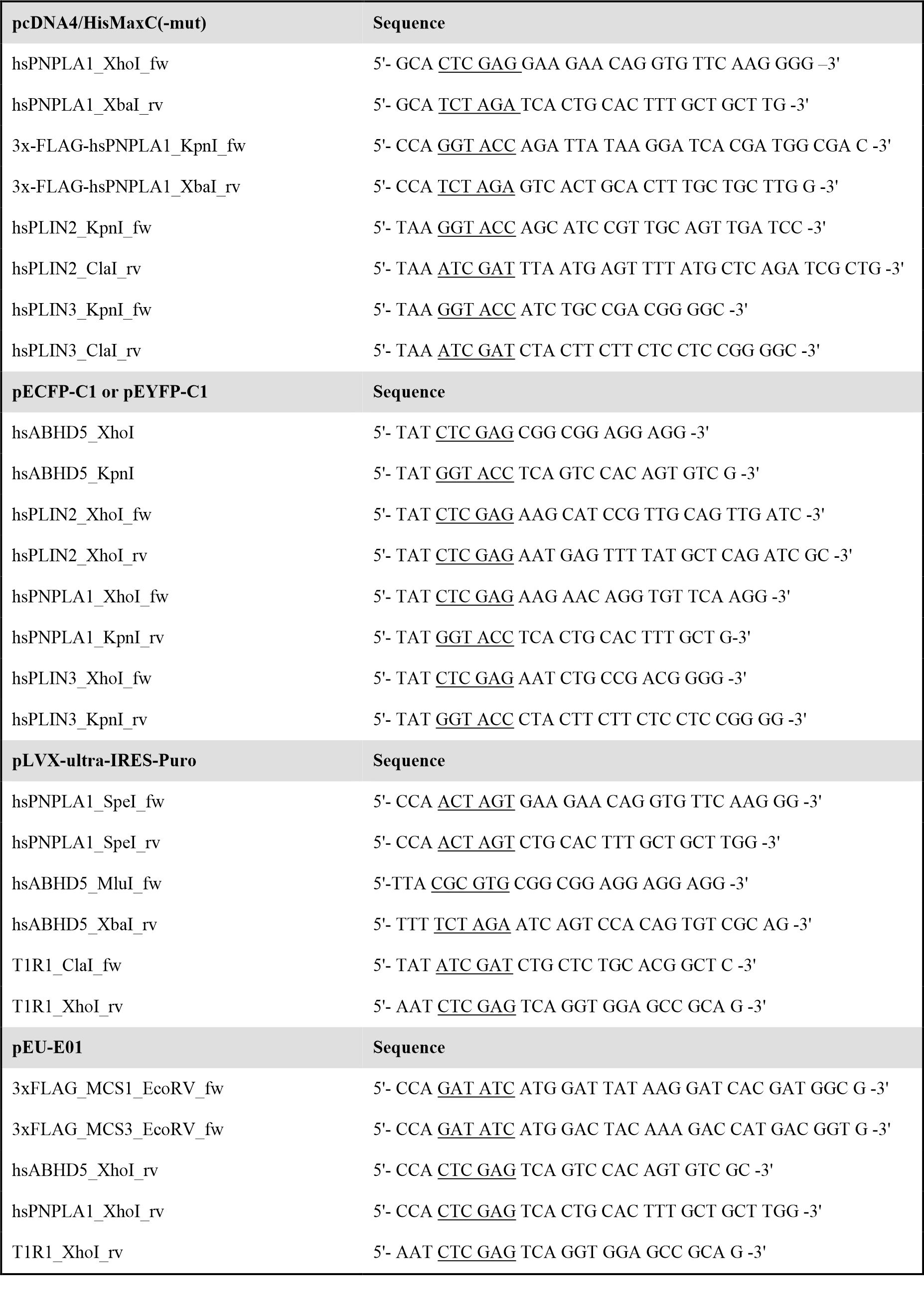


**Supplemental Table 2: Oligonucleotide primers used for site-directed mutagenesis PCRs.** Substituted bases are indicated by lowercase letters.


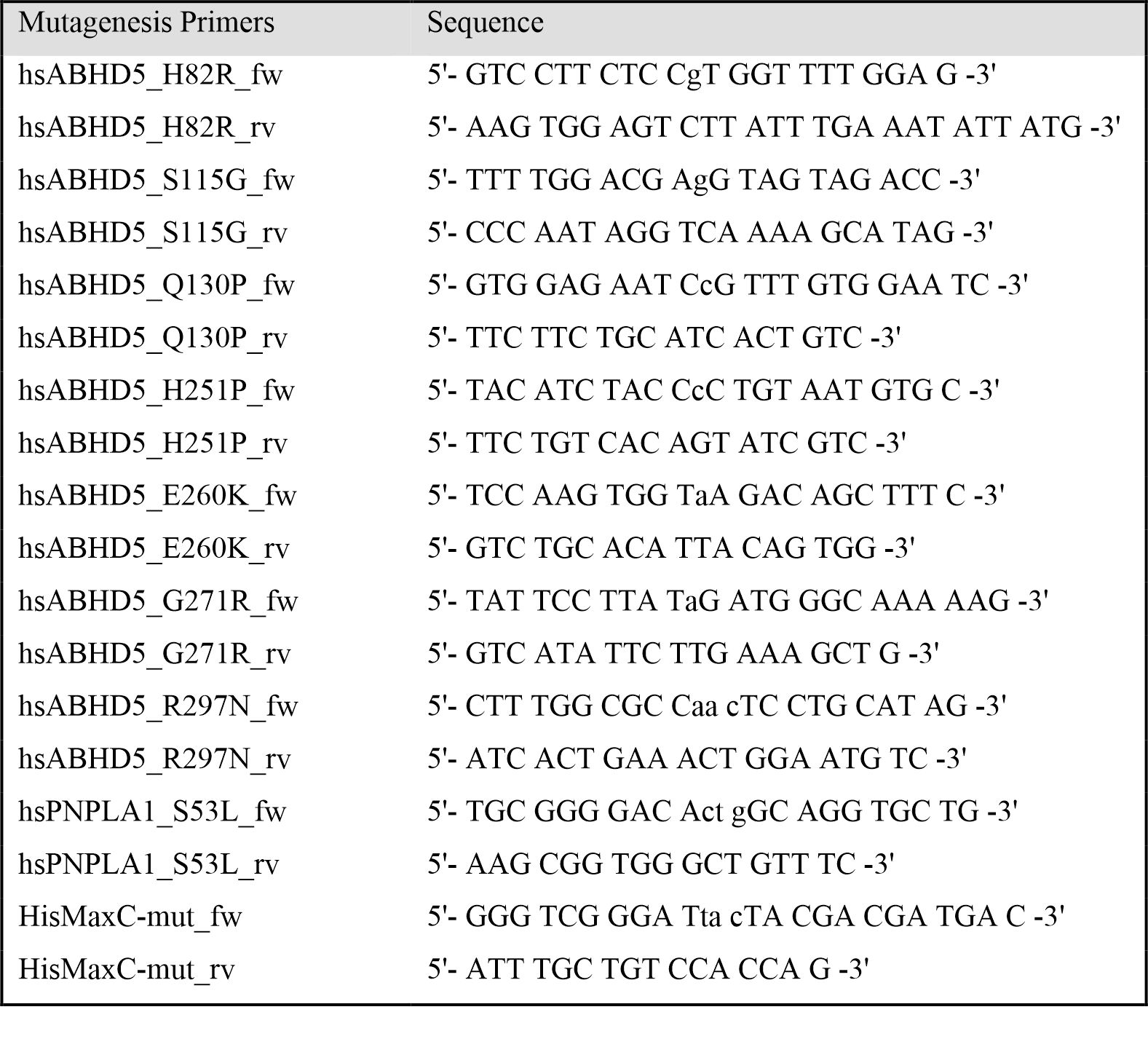


**Supplemental Table 3: Mass spectrometry transitions and corresponding collision energies used for identification of lipid species in targeted triple quadrupole (QQQ) analysis.**
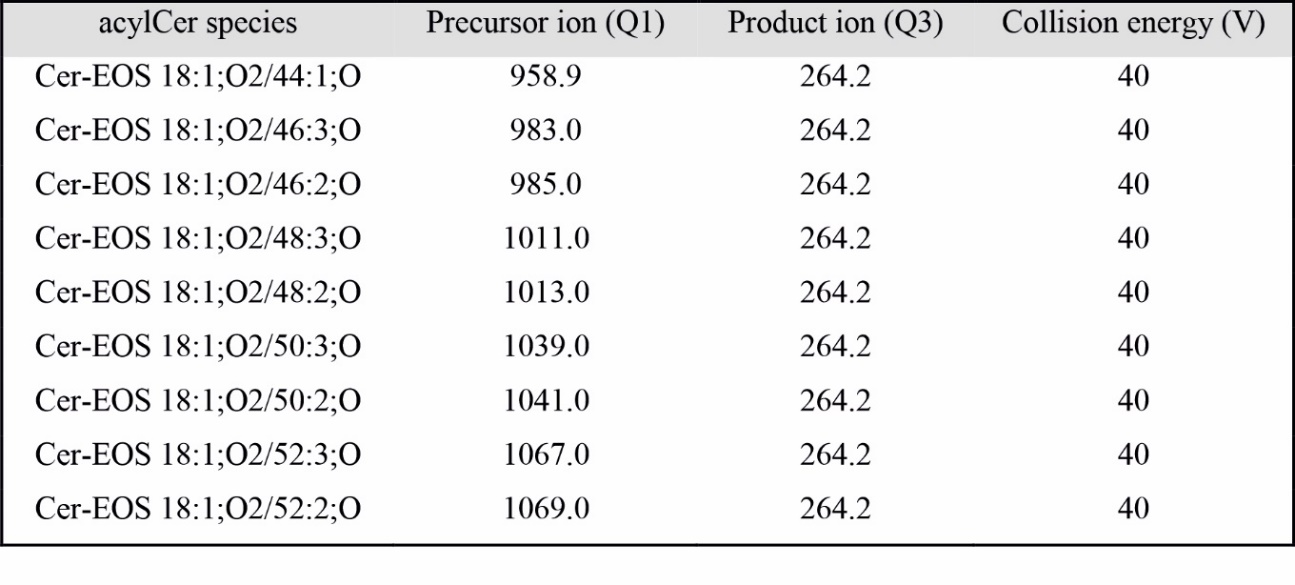


**
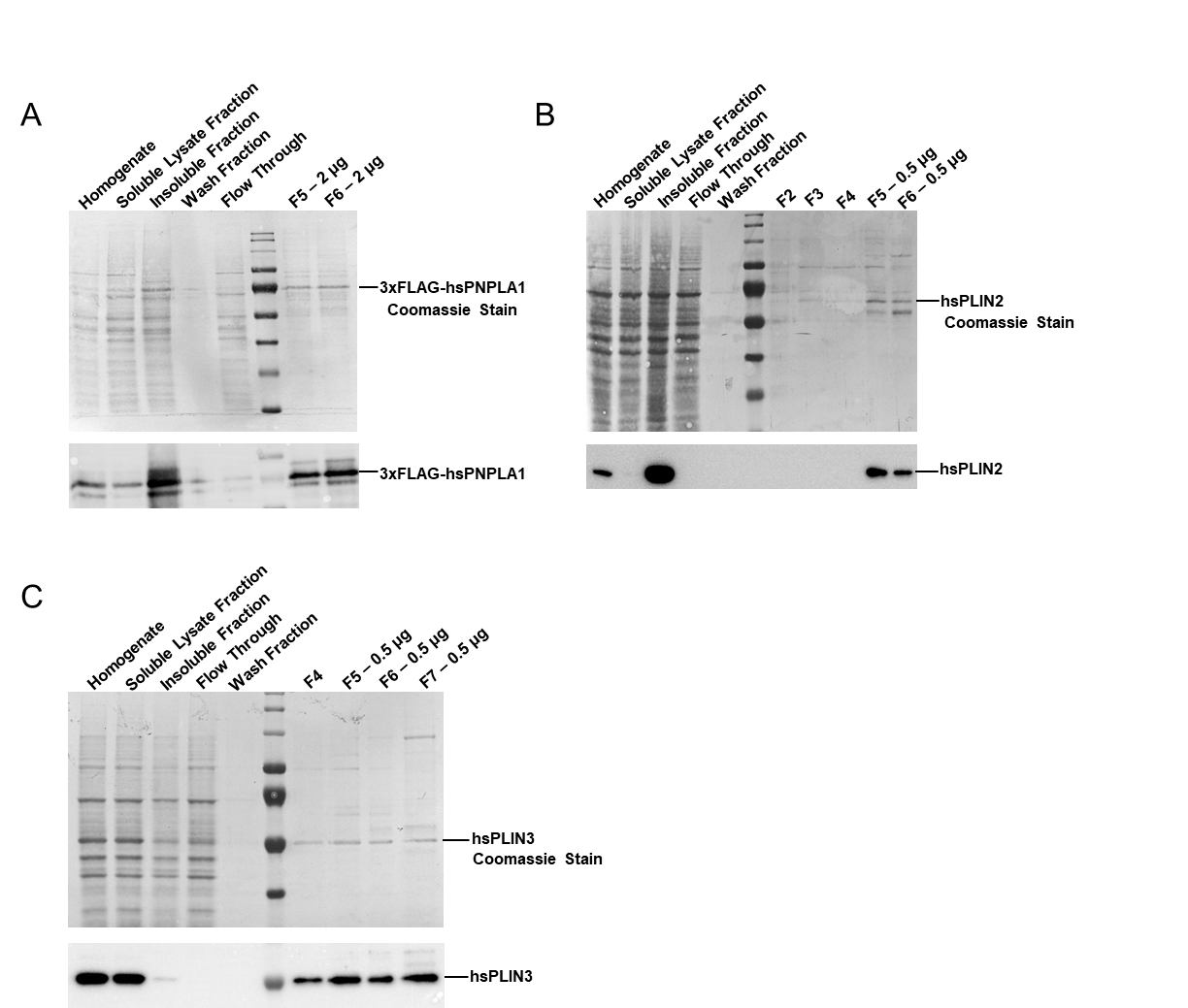
Supplemental Figure 1: Purification of human PNPLA1, PLIN2, and PLIN3 proteins by immobilized metal affinity chromatography (IMAC) using an ÄKTA pure™ system.** A: 3xFLAG-tagged human PNPLA1 protein (3xFLAG-hsPNPLA1) was detected in chromatographic fractions by immunoblot analysis using HRP-conjugated anti-FLAG antibody (lower panel). B: His-tagged human PLIN2 protein (hsPLIN2) was detected by immunoblotting in different chromatographic fractions using anti-6X His tag® antibody and HRP-conjugated anti-mouse secondary antibody (lower panel). C: His-tagged human PLIN3 protein (hsPLIN3) was detected by immunoblot analysis in different chromatographic fractions using anti-6X His tag® antibody and HRP-conjugated anti-mouse secondary antibody (lower panel). A-C: PVDF membranes were stained with Coomassie Brilliant Blue to verify the purity of each fraction (upper panels).


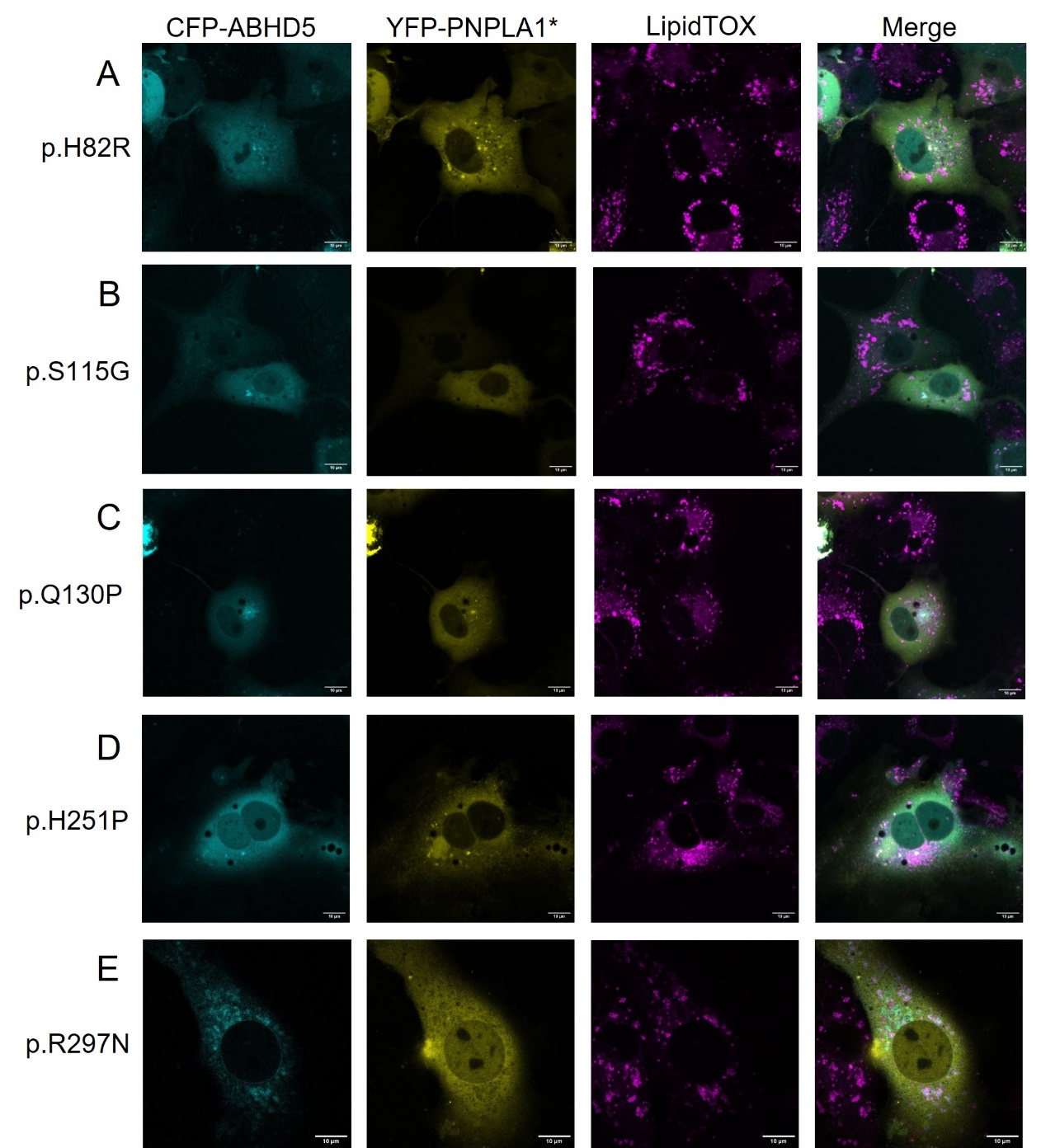


**Supplemental Figure 2: Intracellular localization of a catalytically inactive YFP-tagged PNPLA1 variant (YFP-PNPLA1*) in the presence of CFP-tagged ABHD5 mutants.** A-D: Cells co-expressing YFP-PNPLA1* and the indicated CFP-ABHD5 mutants display increased cytosolic YFP and CFP signals. E: Cells co-expressing YFP-PNPLA1* and CFP-ABHD5 mutant p.R297N exhibit increased CFP signal localized to intracellular lipid droplets, along with increased cytosolic YFP signal. Representative images are shown. Scale bars represent 10 µm.


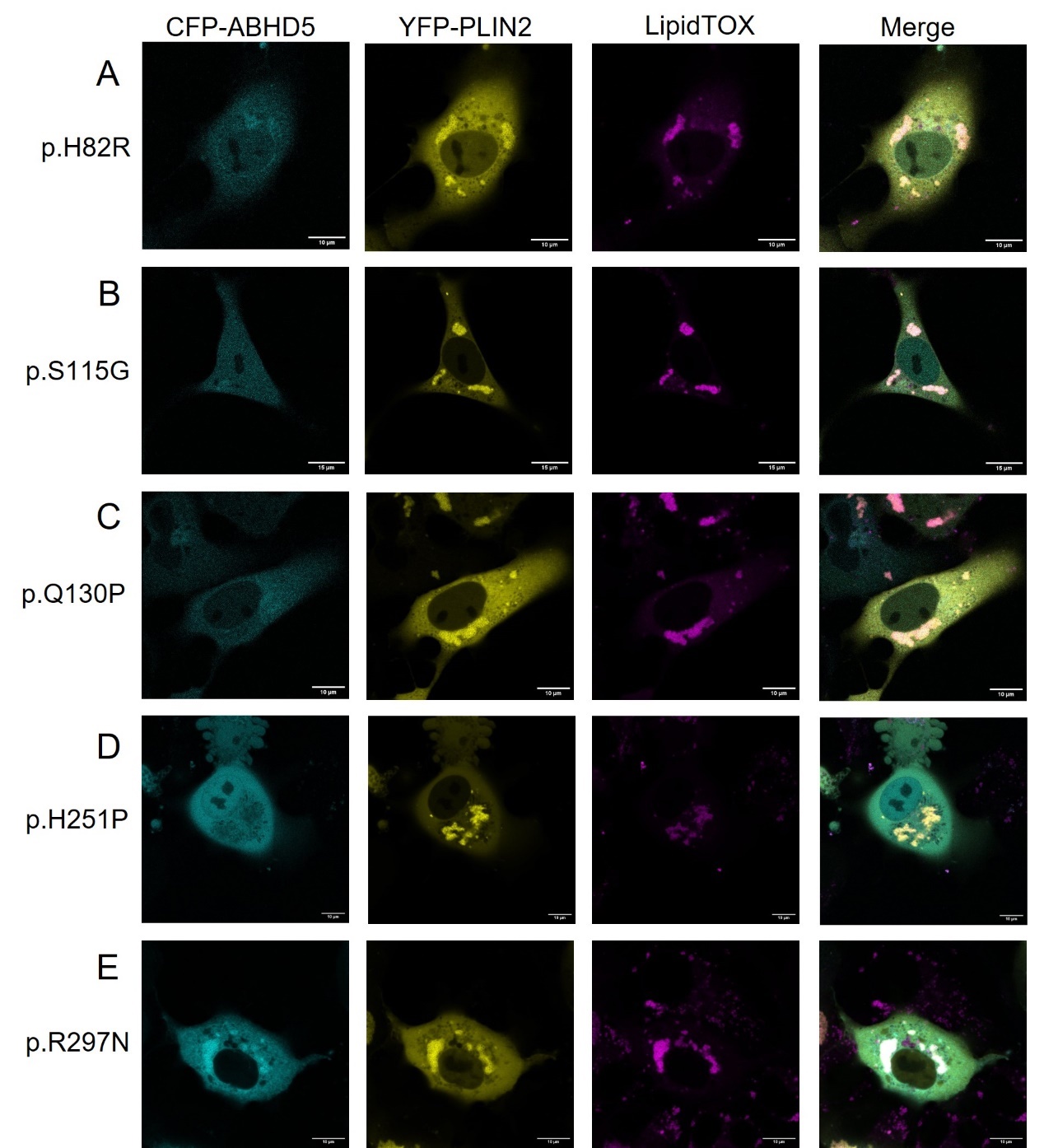


**Supplemental Figure 3: Intracellular localization of CFP-tagged ABHD5 mutants in the presence of YFP-tagged PLIN2.** A-D: Cells co-expressing YFP-PLIN2 and the indicated CFP-ABHD5 mutants show increased YFP signal at intracellular lipid droplets (LDs), while CFP signal is distributed throughout the cytosol. E: Cells co-expressing YFP-PLIN2 and CFP-ABHD5 mutant p.R297N exhibit increased CFP and YFP signals localized to intracellular LDs. Representative images are shown. Scale bars represent 10 µm.


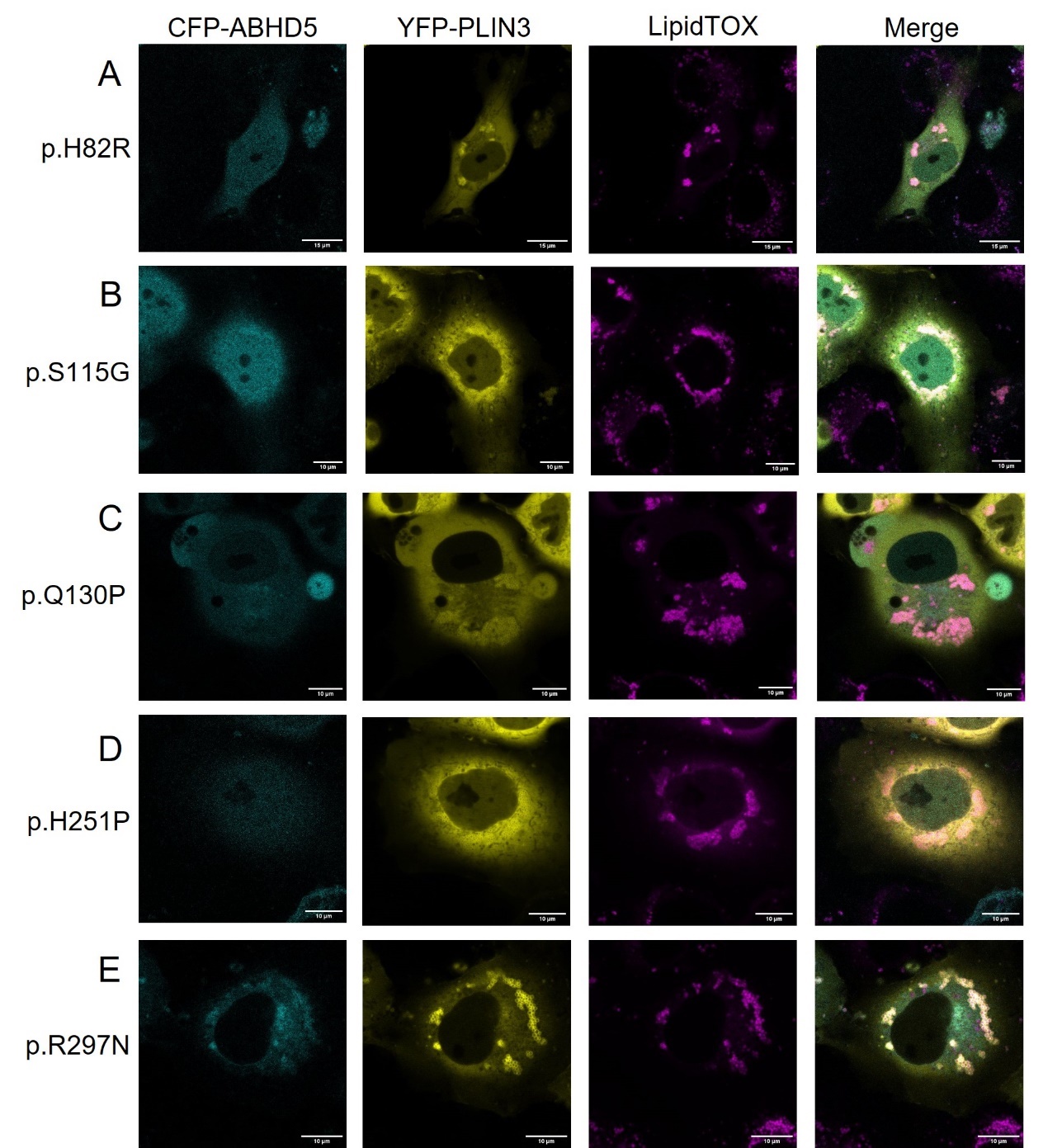


**Supplemental Figure 4: Intracellular localization of CFP-tagged ABHD5 mutants in the presence of YFP-tagged PLIN3.** A-D: Cells co-expressing YFP-PLIN3 and the indicated CFP-ABHD5 mutants show increased YFP signal at intracellular lipid droplets (LDs), while CFP signal is distributed throughout the cytosol. E: Cells co-expressing YFP-PLIN3 and CFP-ABHD5 mutant p.R297N exhibit increased CFP and YFP signal localized at intracellular LDs. Representative images are shown. Scale bars represents 10 µm.


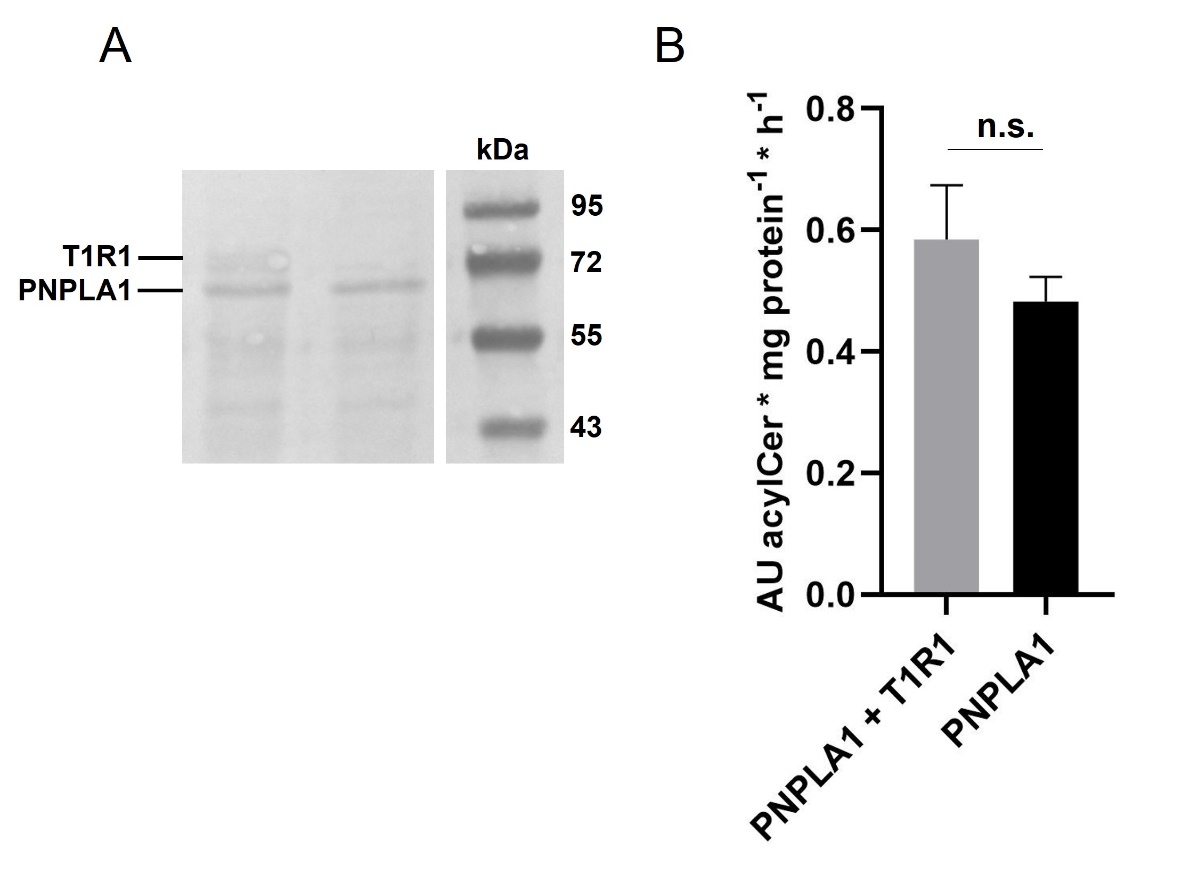


**Supplemental Figure 5: Taste receptor type 1 member 1 (T1R1) does not affect PNPLA1 enzyme activity.** A: Expression analysis indicates similar protein amounts of PNPLA1 in the presence or absence of T1R1 in purified proteoliposomes using Coomassie Blue staining. B: PNPLA1-mediated acylCer synthesis activity of proteoliposomes in the presence or absence of T1R1. AcylCer levels were determined using UHPLC-MS/QQQ. Data are presented as means of quadruplicates + SD and are representative of three independent experiments. Statistical significance was determined using a two-tailed Student’s *t*-test (n.s., *P* > 0.05).
